# A One Stone Three Birds Paradigm of Photon‐Driven Pyroptosis Dye for Amplifying Tumor Immunotherapy

**DOI:** 10.1002/advs.202409007

**Published:** 2025-01-13

**Authors:** Shuang Zeng, Chen Chen, Dan Yu, Maojun Jiang, Xin Li, Xiaosheng Liu, Zhihan Guo, Yifu Hao, Danhong Zhou, Heejeong Kim, Heemin Kang, Jingyun Wang, Qixian Chen, Haidong Li, Xiaojun Peng, Juyoung Yoon

**Affiliations:** ^1^ State Key Laboratory of Fine Chemicals Dalian University of Technology 2 Linggong Road, Hi‐tech Zone Dalian 116024 China; ^2^ School of Bioengineering Dalian University of Technology 2 Linggong Road, Hi‐tech Zone Dalian 116024 China; ^3^ Shanghai Institute of Materia Medica Chinese Academy of Sciences Shanghai 201203 China; ^4^ Shanghai Changzheng Hospital Naval Medical University Shanghai 20000 China; ^5^ School of Chemistry Dalian University of Technology Dalian 116024 China; ^6^ Department of Chemistry and Nanoscience Ewha Womans University Seoul 03760 South Korea; ^7^ Department of Materials Science and Engineering Korea University Seoul 02841 South Korea; ^8^ Innovation Center of Yangtze River Delta Zhejiang University Jiaxing 314100 China

**Keywords:** hemicyanine dyes, immunogenic cell death, immunotherapy, photodynamic therapy, photon‐driven pyroptosis

## Abstract

Activating the pyroptosis pathway of tumor cells by photodynamic therapy (PDT) for immunogenic cell death (ICD) is considered a valid strategy in pursuit of antitumor immunotherapy, but it remains a huge challenge due to the lack of reliable design guidelines. Moreover, it is often overlooked that conventional PDT can exacerbate the development of tumor immunosuppressive microenvironment, which is apparently unfavorable to clinical immunotherapy. The endoplasmic reticulum's (ER) pivotal role in cellular homeostasis and its emerging link to pyroptosis have galvanized interest in ER‐centric imaging and therapeutics. Herein, using the targeted group‐assisted strategy (TAGS), an intriguing cyclooxygenase‐2‐targeted photodynamic conjugate, **Indo‐Cy**, strategically created, which exploits the enzyme's overabundance in the tumoral ER, especially under proinflammatory hypoxic conditions. This conjugate, with its highly precise ER imaging, embodies a trifunctional strategy: i) innovating an electron transfer mechanism, converting the hemicyanine moiety into an oxygen‐independent type I photosensitizer, thereby navigating around the hypoxia constraints of traditional PDT; ii) executing precise ER‐targeted PDT, amplifying caspase‐1/GSDMD‐mediated pyroptosis for ICD; 3) attenuating immunosuppressive pathways by inhibiting cyclooxygenase‐2 downstream factors, including HIF‐1α, PGE2, and VEGF. **Indo‐Cy**'s multimodal approach potently induces in vivo tumor pyroptosis and bolsters antitumor immunity, underscoring cyclooxygenase‐2‐targeted dyes' potential as a versatile oncotherapeutics.

## Graphical abstract

The proposed **Indo‐Cy** conjugate emerges as an avant‐garde molecular entity, adeptly surmounting the recalcitrant hypoxic and immunosuppressive tumor microenvironments to heighten the cytocidal impact of photon‐driven pyroptosis.

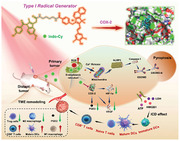

## 1 Introduction

The endoplasmic reticulum (ER), the largest membrane‐bound organelle in cells, plays a central role in regulating fundamental cellular processes.^[^ ^1^ ^]^ It is instrumental in the synthesis and transport of proteins, the maintenance of calcium homeostasis, and the metabolism of lipids processes that are vital for preserving the integrity and proper functioning of the cells.^[^ ^2^ ^]^ Perturbations in ER function, manifesting as ER stress, are etiological in a spectrum of pathologies, notably oncogenesis and neurodegeneration.^[^ ^3^ ^]^ The ER's role in cell death pathways positions it as a strategic target for oncotherapeutic intervention, which can activate a variety of cell death mechanisms through distinct downstream signaling cascades.^[^ ^4^ ^]^

Photosensitizers, fluorescent dyes with dual diagnostic and therapeutic roles, are key components of photodynamic therapy (PDT), a minimally invasive modality noted for its precise spatiotemporal controllability.^[^ ^5^ ^]^ PDT has been verified to induce pyroptosis, a programmed cell death that facilitates the release of tumor‐associated antigens, thereby enhancing the immune response through activation, proliferation, and infiltration of antigen‐specific T cells.^[^ ^6^ ^]^ Within the spectrum of clinical photodynamic therapies, the majority of photosensitizers in current use are classified as Type II, which facilitate the conversion of triplet oxygen (^3^O_2_) to singlet oxygen (^1^O_2_) through an energy transfer process.^[^ ^5^ ^]^ However, the therapeutic efficacy of these photosensitizers is critically dependent on the availability of oxygen, which is often scarce in the hypoxic microenvironment characteristic of solid tumors.^[^ ^7^ ^]^ This limitation renders Type II photosensitizers less effective in inducing pyroptosis, a form of programmed cell death, during standard PDT protocols. In contrast, Type I photosensitizers, which mediate their effects through electron transfer reactions, have shown promise in mitigating the effects of hypoxia on PDT outcomes.^[^ ^8^ ^]^ The ROS produced by these photosensitizers can engage in redox reactions, such as the Haber‐Weiss and Fenton reactions, which are pivotal for the regeneration of oxygen within the tumor microenvironment.^[^ ^9^ ^]^ Therefore, the imperative for the development of novel Type I photosensitizers is evident, as they hold the potential to enhance the initiation of pyroptosis in hypoxic tumor cells, thus offering a significant advancement in the treatment of solid tumors that are refractory to conventional PDT. Nonetheless, our recent research identified that the occurrence of pyroptosis is more pronounced when PDT at the specific sub organelles, e.g. ER and mitochondria.^[^ ^10^ ^]^ Very recently, pyroptosis was determined to cause an overabundance of prostaglandin E2 (PGE2), a cyclooxygenase 2 (COX‐2) metabolite, which may foster an immunosuppressive tumor microenvironment (TME), a factor that could impede immunotherapeutic outcomes.^[^ ^11^ ^]^

COX‐2, predominantly situated in ER, is frequently overexpressed in inflammatory and oncogenic microenvironments while remaining quiescent in healthy cells.^[^ ^12^ ^]^ Its transcription is clarified to be upregulated by inflammatory cytokines and hypoxia‐inducible factor (HIF‐1α).^[^ ^13^ ^]^ The downstream metabolite, PGE2, plays a pivotal role in sculpting an immunosuppressive antitumor microenvironment, implicated in dampening T cell activation and inducing a shift in macrophage polarization toward the immunosuppressive M2 phenotype.^[^ ^14^ ^]^ Further complicating matters, the oxygen depletion inherent to the traditional PDT is postulated to stimulate COX‐2 expression, thereby perpetuating the production of immunosuppressive PGE2. Moreover, pyroptosis‐induced release of proinflammatory cytokines can further amplify COX‐2 levels, potentially attenuating antitumor immune responses. Hence, the interrelated molecular signalings present formidable obstacles to the efficacious immunotherapy of tumors, particularly in pertinent to pyroptosis.

Indomethacin (**Indo**) is an analgesic in clinical practice for the treatment of a variety of inflammatory conditions because of its ability to suppress PGE2 synthesis through inhibition of COX‐2.^[^ ^15^ ^]^ Leveraging **Indo**'s affinity and inhibitory effects on COX‐2, numerous multifunctional chemotherapeutics and fluorescent probes have been engineered, enhancing their potential in targeted oncotherapies.^[^ ^16^ ^]^ Therefore, to achieve potent antitumor immunity, there is an urgent need for the development of a novel dye that can overcome hypoxia, efficiently activate tumor cell pyroptosis under hypoxic conditions, and concurrently inhibit COX‐2 activity to reconfigure the tumor immunosuppressive microenvironment, ultimately contributing to amplified immunogenic effects of PDT. To our knowledge, the development of an appropriate dye embodying this strategy has not yet been reported but holds significant promise in advancing photon‐induced immunotherapy.

**Scheme 1:**
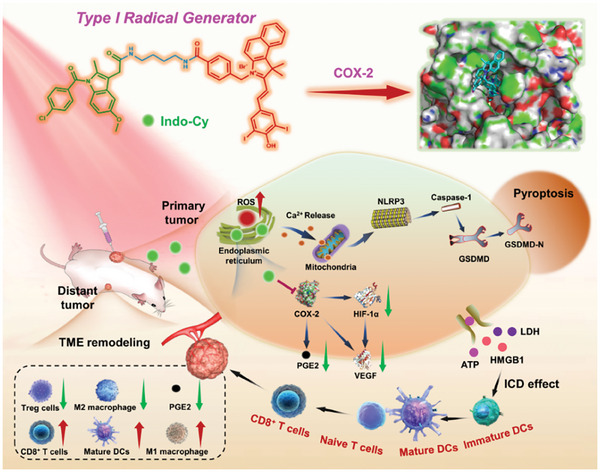
Schematic illustration depicting the molecular mechanisms of **Indo‐Cy** for remodeling the tumor immunosuppressive microenvironment and photodynamic cancer therapy by activating tumor cell pyroptosis.

In this research, we proposed a targeted group‐assisted strategy (TGAS) in the design of Type‐I pyroptotic dye, **Indo‐Cy**, through the covalent linkage of non‐steroidal anti‐inflammatory **Indo** with hemicyanine photosensitizing moiety (**Scheme** **1**). This novel conjugate is envisioned to elicit pyroptosis in cancer cells and concurrently reconfigure the tumor‐associated immune microenvironment. Notably, **Indo‐Cy** undergoes pronounced electron transfer upon excitation to its triplet state, culminating in the formation of a reactive double radical species (In^+^˙‐B‐Cyˉ˙). This initiates an electron transfer pathway with the proximal substrates, yielding hydroxyl radicals (•OH) and superoxide anions (O_2_^•−^), thereby endowing the dye with distinct anti‐hypoxia capabilities. Capitalizing on the ER enrichment of COX‐2,^[^ ^17^ ^]^ **Indo‐Cy** preferentially accumulates in the ER, facilitated by the **Indo** component, enabling precise ER imaging. Under hypoxic conditions typically encountered in PDT, **Indo‐Cy** induces potent ER perturbation, activating a pyroptotic cascade. This pyroptosis is complemented by the immunogenic attributes of cell death, which serve to escalate the recruitment and activation of antigen‐specific T lymphocytes. Moreover, the **Indo** component exerts the targeted inhibition of intracellular COX‐2 activity, attenuating the downstream signaling of pro‐tumorigenic factors such as HIF‐1α, PGE2, and VEGF. This intervention is believed to result in a marked reduction of immunosuppressive T cells and a concomitant upsurge in antitumor M1 macrophages within the tumor microenvironment. The synergistic interplay between pyroptosis induction and immunosuppressive microenvironment modulation by **Indo‐Cy** facilitates the phenotypic transition of “hot” tumors. Hence, this dual‐strategy approach could fully leverage the immunostimulatory effects of pyroptosis, thereby enhancing antitumor efficacies. As such, the primary tumors are subjected to potent PDT‐induced pyroptosis, with distal tumors also poised for immunologic clearance via the proposed immunostimulatory paradigm. This TGAS, tailored for the targeted inhibition of COX‐2, is capable of managing the multifaceted challenges inherent in cancer therapy, thereby illuminating the promising horizons for COX‐2‐targeted photodynamic dyes in the realms of diagnostic imaging and therapeutic intervention.

## 2 Results and Discussion

### 2.1 Photophysical Properties of Photosensitizers **Cy** and **Indo‐Cy**

**Figure 1:**
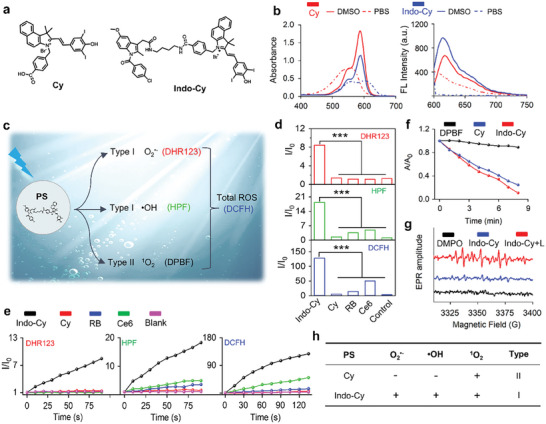
a) The molecular structure of photosensitizers **Cy** and **Indo‐Cy**. b) Absorption and fluorescence spectra of **Cy** and **Indo‐Cy** (10 µm, λ_ex_ = 580 nm) in DMSO and PBS, respectively. c) Schematic diagram of different ROS indicators. d) Fluorescence intensity of DHR123, HPF, and DCFH containing a variety of photosensitizers (10 µm) under photoirradiation, respectively. e) Comparison of the intensity changes of DHR123 and DCFH at 525 nm, HPF at 515 nm as a function of irradiation periods with a variety of photosensitizers, respectively. f) Comparison of the intensity changes of DPBF at 415 nm as a function of irradiation periods with **Cy** and **Indo‐Cy**, respectively. g) EPR signals of DMPO in the presence of **I** **ndo‐Cy**. h) Comparison of the types of ROS produced by **Cy** and **Indo‐Cy**.

The compounds (**Figure** **1**a) were characterized by NMR spectroscopy (^1^H and ^13^C) and high‐resolution mass spectrums (HRMS) (Figures S2–S7, Supporting Information). First, the absorption as well as the fluorescence emission profiles of the photosensitizers of **Cy** and **Indo‐Cy** were examined in solvents. As shown in Figure 1b, the characteristic peaks of UV absorption and fluorescence emission of **Cy** and **Indo‐Cy** in DMSO are generally comparable. The two photosensitizers have a maximum UV absorption at 585 nm and a maximum fluorescence emission at 615 nm, yet contrasting spectral properties were observed in PBS. The UV absorption and fluorescence emission spectra of **Cy** in PBS were subjected to a significant blueshift, with a maximum absorption at 554 nm and a maximum fluorescence emission at 600 nm. The photosensitizer of **Indo‐Cy** displayed dual absorption at 555 and 600 nm, but no significant fluorescence emission could be observed in PBS. This contrast implies that the pathways in electron transition in an aqueous solution are dissimilar if **Indo** is linked with **Cy**. A plausible reason for the observed contrast could be related to the formation of a donor‐bridge‐acceptor (D‐B‐A) structure.

Prior to investigating our proposed dual‐component conjugate of **Indo‐Cy**, the photosensitizing properties of its precursor compound, **Cy** were also investigated. The capacity of **Cy** to produce O_2_^•−^ and •OH was examined in PBS using the specific fluorescent probes DHR123 and HPF (Figure 1c), respectively. As shown in Figures S8 and S9 (Supporting Information), neither O_2_^•−^ nor •OH were produced by **Cy** despite excitation at 580 nm. In contrast, the ^1^O_2_ production of **Cy** was observed (Figure S10a, Supporting Information), as evidenced by the significant decrease in the absorption of the ^1^O_2_ probe (DPBF). These results clearly revealed parental **Cy** as a representative Type II photosensitizer. Furthermore, to quantitatively measure the ability of **Cy** to produce ^1^O_2_, the commercial photosensitizer Rose Bengal (RB, a type II photosensitizer) was used as a control. As shown in Figure S10b (Supporting Information), RB exhibited drastically higher ^1^O_2_ production. DPBF was completely consumed under the same excitation intensities within 30 s. The ^1^O_2_ yield of RB was 0.76, and the ^1^O_2_ yield of **Cy** was calculated to be ≈0.021. To this end, the above results indicate that the precursor photosensitizer of **Cy** is not an outstanding photosensitizer; moreover, its photochemical product is only ^1^O_2_.

However, upon linkage with **Indo**, **Indo‐Cy** appeared to be an excellent photosensitizer not only in terms of the yield of the generated reactive oxygen species (ROS) but also in view of the diversity of the produced ROS. As shown in Figures 1d,e, in stark contrast to **Cy**, **Indo‐Cy** significantly promoted the production of O_2_^•−^ and •OH, as evidenced by the significant increase in the fluorescence intensities of DHR123 and HPF. This specificity in generating free radical ROS was also verified by comparing two classical commercial Type II photosensitizers, RB and Ce6. On the other hand, **Indo‐Cy** and **Cy** were determined to have similar ^1^O_2_‐producing activities (Figure 1f, yield of ^1^O_2_: 0.024). Therefore, our proposed **Indo‐Cy**, due to its appreciable ability to produce free radical ROS, produced remarkably greater amounts of total ROS (detected by DCFH); ≈2.6‐fold greater than that of Ce6, 9.1‐fold greater than that of RB, and 36.6‐fold greater than that of **Cy**. Furthermore, electron paramagnetic resonance (EPR) measurements with the free radical trapping agent DMPO (Figure 1g) also verified the successful production of the free radical signal by **Indo‐Cy**. Hence, these results validated our proposed **Indo‐Cy** as an excellent Type I photosensitizer that can simultaneously produce two types of free radical ROS (Figure 1h), which implies its potential for potent PDT even in a hypoxic microenvironment.

### 2.2 Type I Photosensitization Mechanism of the Dual Component Conjugate of **Indo‐Cy**

**Figure 2:**
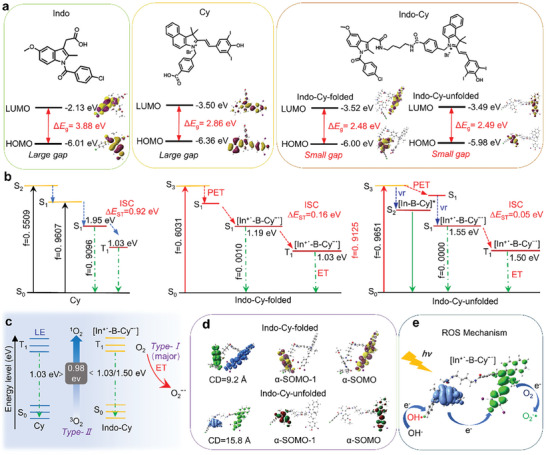
a) Frontier molecular orbitals and energies (eV) from DFT calculations of **Indo**, **Cy,** and **Indo‐Cy** in their ground state (S_0_). b) Calculated lowest excited singlet (S_1_) and triplet energy (T_1_) levels of **Cy** and **Indo‐Cy** in different conformations. c) Schematic diagram of the mechanism by which **Cy** and **Indo‐Cy** generate different ROS through energy transfer and electron transfer, respectively. d) Charge density difference and single electron occupied molecular orbitals in the T_1_ state of **Indo‐Cy‐folded** and **Indo‐Cy‐unfolded**, respectively. e) Schematic diagram of the mechanism by which **Indo‐Cy** generates ROS in solution.

To elucidate the molecular basis for the observed transformation from Type II to Type I photosensitization when coupling **Indo** and **Cy** (**Indo‐Cy**), we performed density functional theory (DFT) and time‐dependent density functional theory (TD‐DFT) calculations for **Indo**, **Cy**, and **Indo‐Cy**. First, after optimizing the structure of **Indo‐Cy**, two limit conformations of **Indo‐Cy** were obtained in aqueous solution, namely, the folded conformation (**Indo‐Cy‐folded**) and the unfolded conformation (**Indo‐Cy‐unfolded**). As shown in **Figure** **2**a, according to the calculation results of the orbital energy level diagrams, after the covalent linkage of **Indo** and **Cy** via alkane chain, most of the LUMO of **Indo‐Cy** is localized on the electron acceptor (i.e., the **Cy** group), while the HOMO is localized on the electron donor (i.e., the **Indo** group). Moreover, this linkage also causes the difference in the HOMO‐LUMO orbital energy levels (ΔE) of **Indo‐Cy** to be smaller than those of both **Indo** and **Cy**. Hence, it becomes favorable for the molecules to undergo electron transfer upon photoexcitation, which is advantageous for the Type I photosensitization pathway based on the electron transfer mechanism for the production of radical ROS.

In principle, the type and efficiency of ROS production by photosensitizers are known to be related to the following three major factors: 1) the intersystem scattering (ISC) rate of the molecules from the excited singlet state (S_1_) to the excited triplet state (T_1_); 2) whether the energy level difference between the T_1_ state and the ground state (S_0_) meets the energy required for the conversion of the triplet oxygen into a singlet oxygen molecule; and 3) whether the molecules in the T_1_ state are able to undergo effective electron transfer and thus produce free radical ROS. First, according to the ISC rate (*K_ISC_*) equation:

(1) $$K_{ISC}\propto {\left(T_{1}\left|H_{\mathrm{SO}}\right|S_{1}\right)}^{2}/{\left(\Delta E_{S1-T1}\right)}^{2}$$

where (T_1_│H_so_│S_1_) is the matrix element of spin‐orbit coupling and ΔE_S1‐T1_ is the energy difference between the lowest excited singlet state (S_1_) and the lowest excited triplet state (T_1_). A smaller ΔE_S1‐T1_ can increase the ISC rate (*K_ISC_*), which is more favorable for enhancing the efficiency of ROS production. Therefore, according to the calculation results in Figure 2b, after the π–π* electron jump, **Cy** only undergoes ISC from S_1_ to T_1_, for which ΔE_S1‐T1_ is 0.92 eV. Since there are two conformations of **Indo‐Cy**, the same analysis was carried out independently for each of **Indo‐Cy**’s two limiting conformations. The subsequent results show that irrespective of **Indo‐Cy** conformation, the HOMO orbital energy level of the electron donor in **Indo‐Cy** is higher than that of the HOMO orbital in the part of the **Cy** moiety.

As a result, intramolecular PET will occur and the ISC pathway from S_1_ to T_1_ can occur when the **Cy** group is photoexcited. Notably, the energy difference ΔE_S1‐T1_ are 0.16 and 0.05 eV, respectively, for the folded and unfolded conformations of **Indo‐Cy**’s. This indicates that **Indo‐Cy** generates ROS effectively under light excitation because of its exceptionally high ISC efficiency. Additionally, there is rivalry between ISC and radiative transition. The transition of S_1_ back to the S_0_ state in the solitary **Cy** is permissible, as can be shown from the vibronic intensity calculation (f = 0.9096). This suggests that some of the energy of the **Cy** will be dissipated as fluorescence, which will reduce the production of ROS. With respect to **Indo‐Cy‐folded**, the electronic transition that has the largest absorption wavelength following light stimulation for **Indo‐Cy‐folded** is a part of the **Cy** group's local excitation (LE) transition (S_3_). Fluorescence quenching occurs as a result of the radiative transition of the S_3_ excited state being prevented by the folding PET effect. The S_1_ exciton is a biradical state [In^+^˙‐B‐Cyˉ˙] with ≈0 oscillator strength (f = 0.0010), which also belongs to the prohibited radiation transition. It is a part of the CT transition from the **Indo** group to the **Cy** group. For **Indo‐Cy‐unfolded**, the largest absorption is also related to the **Cy** group's LE transition (S_2_), but the distance between the **Indo** and **Cy** groups decreases the PET efficiency and produces two distinct types of excitons, i.e., [In‐B‐Cy*] of the LE state and double free radicals [In^+^˙‐B‐Cyˉ˙]* of the CT state.Therefore, when **Indo‐Cy** is in the unfolded conformation, both fluorescence and ROS can be generated.

According to the above results of photophysical properties in solution, the fluorescence emission of **Cy** can be significantly observed in PBS solution, but **Indo‐Cy** has almost no fluorescence emission. It can be reasonably inferred that **I** **ndo‐** **Cy** mainly exists in a folded conformation in aqueous solution, and further calculations also confirm that the folded conformation of **Indo‐Cy** is more stable due to dispersion and π‐π stacking effect (the total energy of the system is ≈100 kcal mol^−1^ lower). However, the conceivable conformations of **Indo‐Cy**, however, should lie roughly between the two limit conformations in biological systems; this is because, when coupled to the target protein, it will take on an unfolded conformation. This may also help to explain why, in later tests, **Indo‐Cy** has been shown to create ROS within cells in addition to fluorescence.

Assuming the photosensitizers are in the excited T_1_ state, there are two ways in which ROS can be produced: either by the electron transfer pathway, which converts the surrounding substrates (water molecules, oxygen molecules, proteins, etc.) into free radicals, or by the energy transfer pathway, which convert the surrounding ^3^O_2_ into ^1^O_2_. Among these, the process of producing ^1^O_2_ based on the energy transfer pathway requires at least 94.5 kJ/mol of energy in order to convert ^3^O_2_ into ^1^O_2_,^[^ ^18^ ^]^ which means that the vertical emission energy of the photosensitizer from the excited T_1_ state to the S_0_ state (ΔE_T1‐S0_) is not less than 94.5 kJ mol^−1^ (i.e., 0.98 eV) so as for sensitizing the surrounding oxygen molecules to become ^1^O_2_ (Figure 2c). Based on the calculation results, it can be seen that the ΔE_T1‐S0_ of **Cy** is 1.03 eV, which satisfied the 0.98 eV required to turn ^3^O_2_ into ^1^O_2_. Nonetheless, due to the limitation of the ISC rate and the occurrence of radiative leaps, the efficiency of producing ^1^O_2_ decreased, which is also consistent with the results of the ROS of **Cy** in PBS (^1^O_2_ yield: 0.021). In contrast, the calculated values of ΔE_T1‐S0_ for **Indo‐Cy** in the two conformations are 1.03 and 1.50 eV, respectively, which should theoretically promote more significant ^1^O_2_ production.

However, an in‐depth analysis of **Indo‐Cy** revealed the formation of a D‐B‐A structure after linking the **Indo** electron‐donating group via an alkane bridge on the **Cy** group. Based on the charge density difference plot of **Indo‐Cy** for different conformations in the T_1_ state (Figure 2d), **Indo‐Cy** could undergo a significant electron transfer during excitation of the triplet state, which has a center‐of‐mass distance of 9.2 and 15.8 Å, respectively. Moreover, this electron‐transfer process prompts the **Indo‐Cy** to form a double radical (In^+^˙‐B‐Cyˉ˙), i.e., the π molecular orbitals of the **Indo** and **Cy** groups each occupy one electron in the T_1_ state (Figure 2d), and this excited state double radical configuration will directly enable the photosensitizer to produce radical ROS via the electron transfer pathway with the surrounding substrate material. To the best of our knowledge, this is the first example in which the excited state configuration of a molecule can be adjusted by rationally designing electron donors and connecting bridges to change the ROS‐producing pathway and promote the transition from a Type II photosensitizer to a Type I photosensitizer.

Based on the above theoretical calculation analysis as well as the results of the previous ROS assay, the specific mechanism of producing free radical ROS from **Indo‐Cy** in solution was further rationalized. As shown in Figure 2e, when **Indo‐Cy** is subjected to excitation, the electron on the **Indo** group is transferred to the **Cy** group to form an excited double radical configuration, at which time the hydroxide ion (OH^−^) originating from the water molecules can transfer the electrons to the electron‐deficient **Indo** group to form the •OH; and the electron‐rich **Cy** group can further transfer the electrons to the oxygen molecules in the water to form the O_2_^•−^. This electron transfer pathway rationally explains the underlying mechanism by which **Indo‐Cy** produces O_2_^•−^ and •OH in aqueous solution.

### 2.3 In Vitro COX‐2 Inhibition and Molecular Docking Mechanism

**Figure 3:**
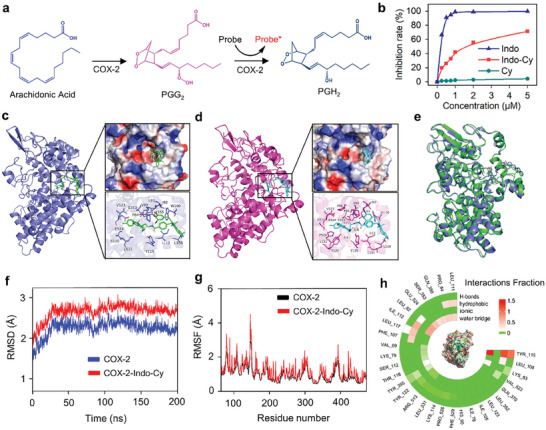
a) Schematic diagram of the detection principle of the COX‐2 inhibitor screening kit. b) Rate of the inhibitory effect of different concentrations of **Indo‐Cy** on COX‐2 activity in solution. c,d) The binding mode of COX‐2 with **Indo‐Cy** before and after MD, respectively. e) The structural superposition of COX‐2 with **Indo‐Cy** before and after MD. f,g) RMSD and RMSF plot during molecular dynamics simulations of COX‐2 with **Indo‐Cy**, respectively. h) The residues that interact with COX‐2 via **Indo‐Cy** during MD simulations.

To verify the validity of the pharmacophore structure obtained by introducing an indomethacin functional group, further studies were conducted to investigate whether the **Indo‐Cy** molecule possesses a COX‐2‐targeted inhibitory effect. We first utilized a COX‐2 inhibitor screening kit to investigate the inhibitory effects of **Indo‐Cy** on the COX‐2 enzyme, and **Indo** was used as a positive control. This kit utilizes the cyclooxygenase activity of COX‐2 to epoxidize substrates such as arachidonic acid to produce intermediates such as PGG_2_, and then utilizes the peroxidase activity of COX‐2 to catalyze intermediates to end products such as PGH_2_ and catalyze the almost non‐fluorescent COX‐2 probe to a strongly fluorescent probe (**Figure** **3**a). Therefore, the inhibition rate can be calculated based on changes in fluorescence signals. As shown in Figure 3b and 1 µm **Indo**, a potent COX‐2 inhibitor, could knock down the activities of COX‐2 by up to 99%. On the other hand, despite the decrease in COX‐2‐inhibiting activity, our proposed **Indo‐Cy** was determined to still exert an appreciable inhibitory effect, the inhibition rate can reach 70% when **Indo‐Cy** at 5 um. Consistent with our expectations, no inhibitory effect was noted for the photosensitizing unit **Cy**.

To explore the molecular binding of **Indo‐Cy** to COX‐2, molecular dynamics (MD) simulations of the complexes of COX‐2 and **Indo‐Cy** were performed for a total of 200 ns. The complex of COX‐2 and **Indo‐Cy** before and after the MD simulation was visualized using PyMOL 2.1 to illustrate the binding pattern of the compound with the protein. According to the binding pattern, the amino acid residues of the protein pocket that bind to the compound can be clearly identified (Figure 3c). A deep pocket is formed in the active site region of COX‐2, and the pocket was characterized to be highly hydrophobic. Hence, the small molecule **Indo‐Cy** matches well with the protein pocket. Moreover, **Indo‐Cy** contains multiple strongly hydrophobic six‐membered rings that can form strong hydrophobic interactions with COX‐2 enzyme pocket sites (LEU‐108, ILE‐112, PRO‐84, TRP‐100, ILE‐92, VAL‐89, LEU‐93, TYR‐115, and TYR‐355) and play important roles in stabilizing **Indo‐Cy**.

After MD simulation, as indicated in Figure 3d, the groove formed by COX‐2 before molecular dynamics simulation became slightly larger, at which time the **Indo‐Cy** compound may better fit into the active site of the protein pocket and form strong hydrogen bonding interactions with the TYR‐115 amino acid, and **Indo‐Cy** is still able to form strong hydrophobic interactions with the surrounding hydrophobic amino acids. As shown in Figure 3e, the binding mode and conformation of **Indo‐Cy** did not change significantly before and after molecular dynamics. When the fluctuation of root‐mean‐square deviation (RMSD) is less than 0.1 nm, the simulation system can be considered to have reached equilibrium. As indicated in Figure 3f, the COX‐2 protein and the COX‐2/**Indo‐Cy** complex began to equilibrate at ≈30 ns, indicating that the structure of the COX‐2/**Indo‐Cy** complex no longer changed.

Root‐mean‐square fluctuation (RMSF) was utilized to monitor the fluctuation level of specific amino acid residues and the overall flexibility changes of the protein molecule. As shown in Figure 3g, due to the binding of the **Indo‐Cy** ligand to the COX‐2, the RMSF values of amino acids such as PRO‐84, PHE‐107, LEU‐108, LEU‐111, ILE‐112, LEU‐117, LEU‐82, and VAL‐523 increased, which once again confirms the binding position of the **Indo‐Cy** ligand through comparison with the binding mode before molecular dynamics simulation and the conformational sampling of the complex at 200 ns (Figure 3e).

The interaction patterns between **Indo‐Cy** and the COX‐2 protein can be divided into four main types: hydrogen bonds, hydrophobic bonds, ionic bonds, and water bridges. As shown in Figure 3h, **Indo‐Cy** interacts well with several amino acids of the protein pocket during the simulation process, such as through strong hydrogen bonding with TYR‐115, and this hydrogen bonding can be present in more than 99% of the molecular dynamic simulation of the entire complex. In addition, **Indo‐Cy** has hydrophobic interactions with the amino acids PRO‐84, PHE‐107, LEU‐108, LEU‐111, ILE‐112, LEU‐117, LEU‐82, and VAL‐523, which play important roles in the targeting of **Indo‐Cy** to anchor the COX‐2 protein pocket.

### 2.4 Subcellular Organelle Colocalization and Intracellular ROS Studies

**Figure 4:**
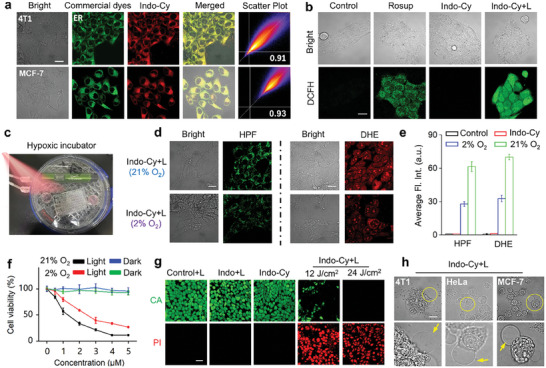
a) The distributions of **Indo‐Cy** (red, λ_ex_ = 580 nm, λ_em_ = 600–700 nm) in 4T1 and MCF‐7 cells by CLSM observation, wherein the endoplasmic reticulum was stained with the commercial dye ER‐Tracker Green (λ_ex_ = 488 nm, λ_em_ = 510–530 nm). Scale bars: 20 µm. b) ROS production in 4T1 cells measured using DCFH‐DA as a fluorescence indicator (green, λ_ex_ = 488 nm, λ_em_ = 510–530 nm). Scale bars: 20 µm. c) Models of the hypoxic intracellular microenvironment. d, e) ROS production in 4T1 cells under normoxic (21% O_2_) and hypoxic (2% O_2_) conditions using DHE and HPF as the O_2_^•−^ and •OH fluorescence indicators, respectively. f) Viabilities of 4T1 cells treated with **Indo‐Cy** under normoxic (21% O_2_) or hypoxic (2% O_2_) conditions. g) Calcein AM (CA, green, 1 µm) and propidium iodide (PI, red, 2 µm) containing fluorescence images of 4T1 cells subjected to different treatments. Red fluorescence (λ_ex_ = 488 nm, λ_em_ = 650–750 nm); green fluorescence (λ_ex_ = 488 nm, λ_em_ = 500–530 nm). h) Representative morphologies of 4T1, HeLa, and MCF‐7 cells treated with **Indo‐Cy** (5 µm) in the presence of excitation (580 nm, 40 mW cm^−2^, 5 min, 12 J cm^−2^). Scale bars: 20 µm. The error bars (*n* = 5) represent means ± SD.

Further therapeutic studies of **Indo‐Cy** at the cellular level were carried out. First, the intracellular distribution of **Indo‐Cy** after cellular uptake was investigated by subcellular organelle co‐localization experiments. Herein, a variety of commercially available organelle dyes were used for the identification of the localization of **Indo‐Cy**. As shown in **Figure** **4**a and Figures S11 and S12 (Supporting Information), **Indo‐Cy** appeared to have a high degree of overlap with the ER, with colocalization coefficients of 0.91 (4T1 cells) and 0.93 (MCF‐7 cells). This high degree of colocalization with the ER could be explained by the increased expression of COX‐2 in the ER and the strong targeting effect of **Indo‐Cy** on COX‐2. Consequently, this highly ER‐targeted function would facilitate the potent photodynamic activation of pyroptosis.^[^ ^19^ ^]^

To verify the adequate PDT activity of **Indo‐Cy** under cellular conditions, the ability of **Indo‐Cy** to produce ROS in the absence and presence of excitation was detected by the fluorescent probe DCFH. In the intracellular ROS production detection experiment, we used untreated cells as a negative control and cells treated with Rosup (a ROS‐generating compound provided by the ROS detection kit produced by Beyotime Biotechnology Co., Ltd) as a positive control. Based on the results of the ROS fluorescence imaging in different cancer cells (Figure 4b; Figure S13, Supporting Information), neither Control nor nonirradiated **Indo‐Cy** could produce ROS in the cells, as evidenced by the minimal fluorescence intensity of the ROS probe DCFH. However, significant fluorescence could be observed in the cells after coincubation with Rosup or cells treated with **Indo‐Cy** upon excitation at 580 nm, which implies that **Indo‐Cy** has excellent PDT effects on the production of ROS after it is internalized by cells.

To verify that the ROS produced by **Indo‐Cy** are consistent with those produced in solution, two other fluorescent probes, HPF and DHE, were used for tracing the intracellular •OH and O_2_^•−^. Aiming to replicate the hypoxic conditions characteristic of solid tumors, we meticulously constructed an in vitro hypoxic cell model utilizing a MIC‐101 chamber from Billups‐Rothenberg (Figure 4c). As shown in Figures 4d,e and Figure S14 (Supporting Information), the specific fluorescence emission of HPF and DHE was observed upon excitation. Importantly, both products can be clearly observed even under anaerobic conditions (2% O_2_), which confirms that **Indo‐Cy** is a Type I photosensitizer and indicates its potential use in hypoxic environments.

Subsequent light/dark toxicity MTT experiments further confirmed that **Indo‐Cy** could cause significant phototoxicity and killing of cells at lower concentrations under both normoxic and hypoxic conditions, with IC_50_ values of 1.109 and 2.494 µm, respectively (Figure 4f). In addition, the phototoxicity of **Indo‐Cy** on tumor cells was also visualized by a CA/PI cell live‐dead staining assay, which showed that the phototoxicity of **Indo‐Cy** on tumor cells was light–dose‐dependent and that the tumor cells were completely killed when the light dose reached 24 J/cm^2^ (Figure 4g).

### 2.5 The Mechanism of Photodynamic Activation of Pyroptosis

Based on the previous report,^[^ ^10a^ ^]^ ER‐targeted PDT can effectively activate the pyroptosis pathway after the photodynamic release of ROS to damage the ER, causing tumor cells to trigger the pyroptosis pathway, which has immunogenic death characteristics. Therefore, we speculate that **Indo‐Cy**, which has dual COX‐2‐inhibiting and ER‐targeting functions, can also effectively activate tumor cell pyroptosis. First, the morphology of different kinds of cancer cells (4T1, HeLa, and MCF‐7) was observed after incubation with **Indo‐Cy** and light treatment. As shown in Figure 4h, the morphological features of pyroptosis were clearly observed in all three cancer cell lines, and there were significant bulges protruding from the cell membrane in all tested cells.

**Figure 5:**
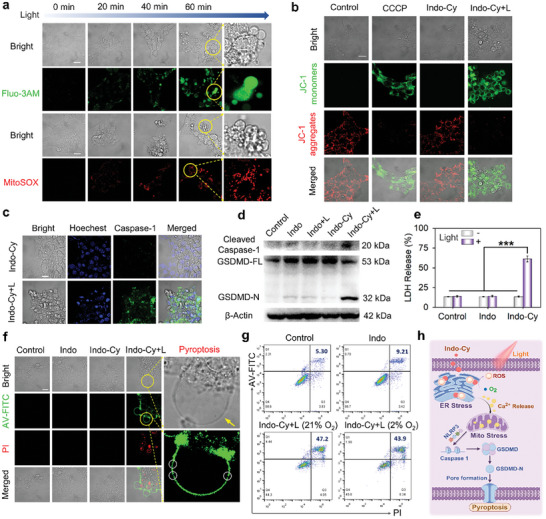
a) Time‐dependent changes in the intracellular calcium concentration and mitochondrial oxidative stress in 4T1 cells treated with **Indo‐Cy** (5 µm) upon excitation at 580 nm (40 mW cm^−2^, 5 min, 12 J cm^−2^). b) Mitochondrial membrane potential assay of 4T1 cells using JC‐1 as a fluorescence indicator. c) Immunofluorescence staining of **Indo‐Cy**‐triggered caspase‐1 activation in 4T1 cells. Scale bars: 20 µm. d) Western blot analysis of the expression of caspase‐1 and the liberated GSDMD in 4T1 cells treated with **Indo‐Cy** (5 µm) in the presence or absence of excitation at 580 nm. e) Quantification of LDH release. f) Annexin V‐FITC/PI costaining assay, red fluorescence of PI (λ_ex_ = 488 nm, λ_em_ = 650–750 nm) and green fluorescence of Annexin V (λ_ex_ = 488 nm, λ_em_ = 510–530 nm). Scale bar: 20 µm. g) Quantitative analysis of Annexin V‐FITC and PI in 4T1 cells treated with **Indo‐Cy** by flow cytometry. h) Schematic illustration of the proposed pyroptosis activation via **Indo‐Cy**. The figure (h) was created by Figdraw. Statistical significances were calculated by via one‐way ANOVA: ^*^ *p* < 0.05, ^**^ *p* < 0.01, and ^***^ *p* < 0.001.

To clarify the molecular mechanism by which ER‐targeted PDT (caused by **Indo‐Cy**) activates pyroptosis, we investigated the release of calcium ions following oxidation of the ER and subsequent mitochondrial overloading with Ca^2+^. Herein, Fluo‐3AM was used as an indicator of Ca^2+^. As shown in **Figure** **5**a, a rapid and progressive increase in the fluorescence intensity of Fluo‐3AM was confirmed in the cell interior upon excitation at 580 nm. Moreover, the cell morphologies were also observed to undergo a progressive transformation, wherein the cells appeared to undergo gradual swelling. In particular, bulge‐like bubbles were observed. Given that the endoplasmic reticulum is one of the storage sites for cytosolic calcium ions,^[^ ^20^ ^]^ the consistent release of Ca^2+^ indicates severe injury to the ER due to the targeted oxidation of the ER by **Indo‐Cy**.

On the other hand, MitoSOX, a probe for mitochondrial superoxide, was used to assess the degree of superoxide production in the mitochondria. As shown in Figure 5a, the fluorescence intensity of MitoSOX increased consistently. In addition, the mitochondrial morphologies appeared to also undergo transformation, e.g. mitochondrial shrinkage from elongated strips to spherical shapes. Most likely, the Ca^2+^ released from ER preferentially accumulates in mitochondria due to the mitochondria‐associated membranes.^[^ ^21^ ^]^ The resulting overloading of Ca^2+^ in mitochondria would lead to abnormal respiratory functions of the mitochondria, thus eliciting excessive production of superoxide.^[^ ^22^ ^]^

Moreover, the results of mitochondrial damage were further verified by the results of the mitochondrial membrane potential assay. CCCP, an inhibitor of oxidative phosphorylation, was used as a positive control for the loss of mitochondrial membrane potential. As shown in Figure 5b, the JC‐1 probe was anchored to the mitochondria as aggregates in the normal cells and in the non‐light group due to the normal mitochondrial membrane potential, which resulted in significant red fluorescence. In contrast, for the CCCP and **Indo‐Cy**+L groups, the JC‐1 probe was almost completely dispersed in the cytoplasm, and a large area of green fluorescence was observed, which suggested that the mitochondrial membrane potential almost completely disappeared after cellular photodynamic therapy. This result also verified that ER‐targeted PDT exacerbates calcium release and thus damages mitochondria.

According to previous studies, ROS produced after mitochondrial injury (mtROS) can activate the inflammatory vesicle NLRP3 thereby further activating Caspase 1 and ultimately promoting the hydrolysis of GSDMD into the pyroptosis executing protein, GSDMD‐N, which triggers pyroptosis by punching holes in the membrane.^[^ ^23^ ^]^ Therefore, the degree of intracellular Caspase 1 activation was first detected using a Caspase 1 fluorescent probe. As shown in Figure 5c, bright Caspase 1 probe fluorescence signals were observed only in cells treated with **Indo‐Cy**, and exposed to light, suggesting that the photodynamic triggering of the pyroptosis pathway by **Indo‐Cy** is most likely achieved by the activation of Caspase 1.

Therefore, the protein expression of cells subjected to different treatments was further verified by immunoblotting. As previously reported, activated Caspase 1 protein could be detected only in cells incubated with **Indo‐Cy** and exposed to light, and a decrease in the amount of full‐length GSDMD protein as well as high expression of the GSDMD‐N‐terminal protein was also observed (Figure 5d). After the GSDMD‐N‐terminal protein perforates the membrane to trigger pyroptosis, the fluidity inside and outside of the cell membrane is enhanced, which leads to leakage of the intracellular contents, which is the main reason why pyroptosis is characterized by immunogenic death. LDH release from within the cell can be used to distinguish pyroptosis from apoptosis. As shown in Figure 5e, a large amount of LDH was detected only in the supernatant of cells in the **Indo‐Cy** light group, indicating that tumor cells undergoing pyroptosis no longer possessed membrane integrity due to the perforation activity of the GSDMD‐N‐terminal protein, leading to leakage of LDH.

In addition to LDH, an AV‐FITC/PI assay was also used to investigate the membrane integrity during the process of pyroptosis. Although AV‐FITC is commonly used to detect phosphatidylserine deposition in the cell membrane during apoptosis, AV‐FITC can also penetrate and bind to phosphatidylserine on the inner side of cell membranes due to the formation of membrane pores during pyroptosis.^[^ ^24^ ^]^ The unique bulging feature of pyroptosis can be used to distinguish the two modes of death under a fluorescence microscope. As shown in Figure 5f, the bulge of the protruding cell can be clearly observed after **Indo‐Cy** light‐triggered cell pyroptosis. The bulge of the membrane has some unoccupied breaks, which are thought to be the hole generated by the GSDMD‐N‐terminal protein in the membrane. Furthermore, it was discovered that PI may enter the nucleus and cause it to glow in tandem with the start of pyroptosis. Therefore, the efficiency of pyroptosis initiation could also be verified by flow‐through detection of AV‐FITC/PI. As shown in Figure 5g, according to the flow‐through results, **Indo‐Cy** can efficiently initiate pyroptosis under both normoxic and anoxic conditions because **Indo‐Cy** is a type I photosensitizer that is active in hypoxic environments. A schematic diagram of **Indo‐Cy** light‐induced tumor cell pyroptosis, which triggers the mitochondrial‐Caspase1‐GSDMD signaling pathway by precisely damaging the ER through PDT, is shown in Figure 5h.

### 2.6 Validation of Pyroptosis‐Induced Immunogenic Cell Death (ICD)

**Figure 6:**
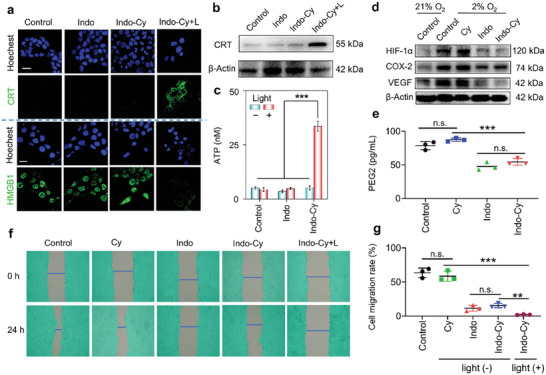
a) Immunofluorescence staining showing the localization of CRT in the membranes and HMGB1 in 4T1 cells. Scale bars: 20 µm. b) CRT after PDT treatment, as determined by Western blot analysis. c) Quantification of ATP release into the extracellular compartment. d) Western blot analysis of HIF‐1α, COX‐2 and VEGF in 4T1 cells subjected to different treatments. e) Quantification of the secretion of PGE2 by ELISA (*n* = 3). Wound healing assay (f) and cell migration rate (g) of 4T1 cells subjected to different treatments. The error bars (*n* = 3) represent means ± SD. Statistical significances were calculated via one‐way ANOVA: n.s. represents no significance, ^*^ *p* < 0.05, ^**^ *p* < 0.01, and ^***^ *p* < 0.001.

To demonstrate that pyroptosis is an immunogenic mode of death that effectively activates immunity, multiple characteristics related to ICD were examined. First, immunofluorescence staining was performed to investigate the extent of calreticulin (CRT) migration to the membranes after photodynamically triggered pyroptosis. As shown in **Figure** **6**a, after **Indo‐Cy** PDT‐induced cell death, intracellular CRT was highly expressed and exposed on the surface of the cell membrane, which could enhance the maturation of DCs and release the “eat me” signal to promote phagocytosis by DCs.^[^ ^25^ ^]^ At the same time, CRT extraction from the cell membranes also confirmed that PDT is essential to provoking the translocation of CRT to the cell membranes (Figure 6b).

In addition, the release of HMGB1 and ATP is also a major feature of ICD. Notably, HMGB1 can be released from the nucleus to the extracellular compartments and recruit immature DCs to tumors, where it binds to cell surface TLR2 to activate the receptor‐mediated signaling pathway, further inducing DCs maturation.^[^ ^26^ ^]^ As shown in Figure 6a, HMGB1 release was also observed by immunofluorescence staining. HMGB1 was highly expressed in the nucleus and highly colocalized with cytosolic dyes in both normal cells and non‐light‐treated cells, whereas HMGB1 almost completely disappeared from the nucleus after **Indo‐Cy**‐triggered pyroptosis. This result suggested that HMGB1 had completely leaked out of the nucleus. Similarly, the increase in the ATP content in the supernatant (Figure 6c) showed that due to the formation of pores in the membrane of the pyroptotic cells, substantial amounts of intracellular ATP were released from the cells. ATP outside a cell is a “find me” signal to rapidly recruit myeloid cells, including DCs, to tumor sites.^[^ ^27^ ^]^ To substantiate the pivotal role of pyroptosis in enhancing the immunogenicity of tumor cells, we conducted an analysis to determine the maturation of DCs, marked by the upregulation of CD80 and CD86, using flow cytometry (as depicted in Figure S15, Supporting Information. The maturation of DCs was ascertained by examining bone marrow‐derived dendritic cells (BMDCs) from Balb/c mice that were co‐cultured with 4T1 tumor cells following PDT. This experimental approach serves to validate the hypothesis that pyroptosis can significantly promote the activation and maturation of DCs, which are crucial for initiating an effective antitumor immune response. In conclusion, **Indo‐Cy**‐induced photodynamic tumor cell death can significantly enhance ICD, which has the potential to activate the body's immune system and thus enhance antitumor immune responses.

### 2.7 Inhibition of the Intracellular COX‐2/PGE_2_ Pathway by **Indo‐Cy**

COX‐2, a highly expressed tumor tissue‐specific enzyme, is involved in a variety of important pathways that promote tumor development, including the regulation of tumor‐associated angiogenesis, the creation of the immunosuppressive microenvironment, and the promotion of cell invasion and metastasis. In addition to COX‐2, PGE_2_, the main downstream product of COX‐2, plays a key role in the regulation of tumor neovascularization, the immunosuppressive microenvironment, cell invasion, and metastasis. In addition to stimulating angiogenesis, PGE_2_, which is secreted extracellularly, can promote the conversion of immature myeloid cells (IMCs) into myeloid‐derived suppressor cells (MDSCs) with immunosuppressive functions, which can inhibit the activation of effector T cells, promote the conversion of M1 macrophages into immunosuppressive M2 macrophages, and induce immunosuppressive T cells (Tregs).^[^ ^28^ ^]^ In particular, Tregs and M2 macrophages have been verified to play important roles in tumor evasion via immune surveillance. Therefore, effective inhibition of the intracellular COX‐2/PGE_2_ pathway is critical for remodeling the immunosuppressive microenvironment and is essential for enhancing pyroptosis‐induced antitumor immunity. Therefore, to further validate the effectiveness of **Indo‐Cy** in targeted COX‐2 inhibition, we investigated the expression levels of COX‐2 and its downstream factors, including HIF‐1α and VEGF, by immunoblotting and immunofluorescence imaging.

Given that COX‐2 is overexpressed in hypoxic solid tumors, the inhibitory effects of **Indo‐Cy** on intracellular COX‐2 under hypoxic conditions were investigated. As shown in Figure 6d, compared to that in normoxic cancer cells, COX‐2 expression in cells incubated with a low oxygen supply was significantly elevated, whereas COX‐2 expression in hypoxic cells was significantly decreased in the presence of **Indo** and **Indo‐Cy**. This result means that **Indo‐Cy**‐containing indomethacin still plays a role in targeting and inhibiting COX‐2 in hypoxic conditions, implying it will interfere with COX‐2 transcription and expression in a hypoxic tumor in vivo. In addition, a number of studies have shown that COX‐2 is positively correlated with the expression of the hypoxia‐inducible factor HIF‐1α in cells.^[^ ^13a,b^ ^]^ Moreover, COX‐2 affects the expression of VEGF through its involvement in the regulation of HIF‐1α expression and thus VEGF expression.^[^ ^13c^ ^]^

Therefore, the effects of **Indo‐Cy** on intracellular HIF‐1α and VEGF protein levels were further investigated. As shown in Figure 6d and Figures S16–S18 (Supporting Information), consistent with the COX‐2 immunoblotting and immunofluorescence results, **Indo** and **Indo‐Cy** also inhibited the intracellular expression of HIF‐1α and VEGF proteins under hypoxic conditions, suggesting that their downstream pathways were significantly inhibited due to the inhibition of COX‐2 expression.

Moreover, the PGE_2_ levels in the cell supernatants were detected by an ELISA kit. As shown in Figure 6e, due to the significant inhibition of COX‐2 by both **Indo** and **Indo‐Cy**, the PGE_2_ level in the cell supernatants was significantly lower than that in the control group and in the **Cy** group treated with photosensitizing motifs alone. This result implies that **Indo‐Cy** has the potential to remodel the immunosuppressive microenvironment of solid tumors. Since COX‐2 is also associated with cell migration, we further verified the effect of COX‐2 inhibition on cell migration by a cell scratch assay. According to the cell migration distance, the cell migration rates of the normal control and **Cy** groups were high, ≈60%, due to the strong migratory ability of these cancer cells (Figures 6f,g). After treatment with **Indo** and **Indo‐Cy**, the cell migration rates significantly decreased to less than 20%, and no cell migration was achieved with further photodynamic treatment. This result demonstrated that COX‐2 also plays a key role in tumor cell migration, which may be related to the involvement of COX‐2 in the regulation of the expression of promigratory proteins, such as VEGF, in cells.

### 2.8 In Vivo Antitumor Efficacy in a 4T1 Tumor Model

**Figure 7:**
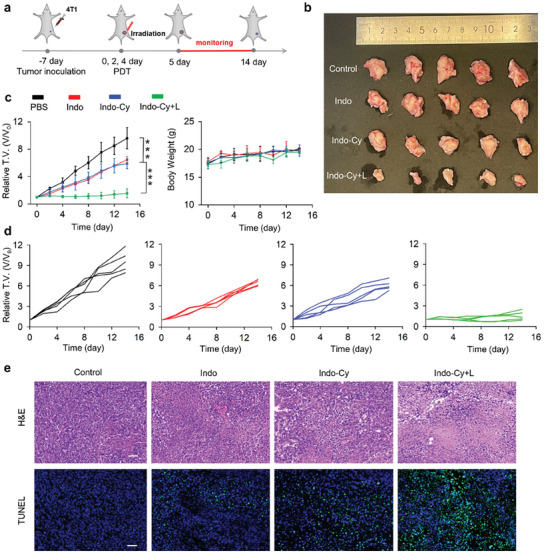
a) Illustration of the therapeutic scheme for the treatment of solid tumors with **Indo‐Cy**. b,c) Relative tumor growth and changes in the body weights of the mice after various treatments. d) Individual tumor volume after various treatments in mice. e) H&E and TUNEL assays of the tumors on Day 14 after different treatments. Scale bars: 50 µm. The error bars (*n* = 5) represent means ± SD. Statistical significances were calculated via one‐way ANOVA: ^*^ *p* < 0.05, ^**^ *p* < 0.01, and ^***^ *p* < 0.001.

Given the stimulation of pyroptosis and targeted inhibition of COX‐2 by **Indo‐Cy**, we were further motivated to utilize **Indo‐Cy** for the treatment of 4T1 tumor‐bearing mice. To ensure that the drug acted effectively on the tumors, **Indo‐Cy** was applied by direct intratumor injection. The tumor volumes of each group were recorded every two days. The therapeutic scheme is summarized in **Figure** **7**a. After the end of the treatment, it was clear that the tumors of the control mice injected with PBS grew most rapidly (Figure 7b). Moreover, the tumor volumes of the mice injected with **Indo** and **Indo‐Cy** were also observed to increase at a relatively rapid rate (despite a slight decrease). Interestingly, the most potent tumor growth suppression was achieved by our proposed **Indo‐Cy**‐based PDT (Figures 7c,d), confirming our strategy for the manufacture of a multifaceted PDT therapeutic agent for improved antitumor therapy. Reassuringly, the average body weights of the **Indo‐Cy**‐treated mice were comparable to those of PBS‐treated mice, suggesting the overall safety of our proposed PDT with **Indo‐Cy**. Furthermore, H&E and TUNEL assays of the excised tumor tissues revealed abundant cell death in response to **Indo‐Cy**‐based PDT, as indicated by the large‐scale vacancies in the tumors (Figure 7e).

To investigate the inhibitory effect of **Indo‐Cy** on tumoral COX‐2, COX‐2, CD31, HIF‐1α, and VEGF expression in the tumor tissue was further analyzed by immunofluorescence staining. As shown in Figure S19 (Supporting Information), the **Indo‐Cy** conjugate in the absence of excitation had a comparable ability to inhibit COX‐2 as that of the **Indo** group alone, and both conjugates significantly inhibited the expression of intratumor COX‐2, CD31, HIF‐1α, and VEGF. This inhibitory effect can counteract the enhanced COX‐2 expression in tumors due to the intensified hypoxia induced by PDT, which suggests the potential of **Indo‐Cy** for remodeling the immunosuppressive tumor microenvironment.

### 2.9 Inhibition of COX‐2 Expression by **Indo‐Cy** Activates Pyroptosis to Remodel the Tumor Microenvironment

The above investigations validated the appreciable ability of **Indo‐Cy** to exert PDT and inhibit COX‐2 in vivo. The ensuing impact on the regulation of antitumor immunity was also studied. A number of previous studies have demonstrated that pyroptosis is a highly immunogenic death pathway that can activate the body's adaptive immune system and stimulate the proliferation of immune T cells for long‐lasting tumor clearance. However, tumors can recruit a variety of immunosuppressive cells to counteract this immunostimulation, thereby limiting the antitumor efficacy of conventional pyroptosis‐initiated immunotherapy.^[^ ^29^ ^]^

**Figure 8:**
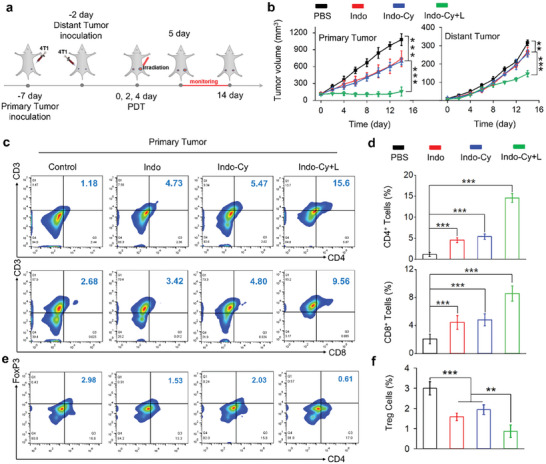
a) Illustration of the therapeutic scheme used for the treatment of primary tumors and distant tumors. b) Changes in primary and distant tumor volumes after the indicated treatments, respectively. c) Representative flow cytometry images of CD4^+^ T cells and CD8^+^ T cells among CD3^+^ tumor‐infiltrating leukocytes in the primary tumor tissues. d) Quantification of the percentages of CD4^+^ T cells and CD8^+^ T cells in primary tumors based on flow cytometry. e) Representative flow cytometry images of Treg cells (CD3^+^CD4^+^Foxp3^+^) in tumor‐infiltrating CD4^+^ T cells in primary tumor tissues. f) Quantification of the percentages of Treg cells in primary tumors based on flow cytometry measurements. The error bars (*n* = 5) represent means ± SD. Statistical significances were calculated via one‐way ANOVA: ^*^ *p* < 0.05, ^**^ *p* < 0.01, and ^***^ *p* < 0.001.

In addition, PGE_2_ in the TME was reported to not only inhibit the antitumor activities of DCs but also facilitate tumorigenesis via Tregs and M2 macrophages.^[^ ^30^ ^]^ Therefore, **Indo‐Cy**, which has a COX‐2‐targeted inhibitory effect, was used as a unique conjugate to reprogram the immunosuppressive tumor microenvironment, aiming to further amplify adaptive immunity enhanced by the photodynamic induction of pyroptosis. To gain insight into the role of **Indo‐Cy** photodynamic immunotherapy in vivo, a mouse primary tumor versus distal tumor model was established for the current study. The therapeutic scheme is summarized in **Figure** **8**a, and it is evident that, in addition to the significant suppression achieved in the primary tumors, PDT by **Indo‐Cy** could also contribute to potent tumor growth inhibition in the distal tumors (Figure 8b).

Further flow cytometry analysis of the percentages of CD8^+^ and CD4^+^ T cells in the primary tumors as well as the distal tumors (Figure 8c,d; Figures S20 and S21, Supporting Information revealed a significant increase in the percentages of CD8^+^ and CD4^+^ T cells in both the primary and distal tumors after PDT treatment. The enhanced intratumoral infiltration of CD8^+^ and CD4^+^ T cells was further demonstrated by immunofluorescence staining of tumor sections (Figure S22, Supporting Information). In contrast to the limited increase in the population of immune T cells after treatment with **Indo‐Cy** (as well as **Indo**) in the absence of excitation, pyroptosis induced by PDT more effectively promoted immune T‐cell maturation, enhanced immune infiltration, and ultimately contributed to stronger antitumor immune responses.

To verify that amplified antitumor immunity was also correlated with alterations in the immunosuppressive microenvironment, the populations of immunosuppressive T cells, as well as M1‐ and M2‐type macrophages, in the primary tumors were measured. As expected, as shown in Figures 8e,f, **Indo‐Cy**, as well as **Indo**, appeared to be capable of reducing the population of immunosuppressive Tregs in tumors despite the absence of light irradiation due to the functional component of **Indo**. Notably, the population of Tregs was found to further decrease upon irradiation. The ratio of M1 to M2 macrophages in tumors also varied significantly (Figure S23, Supporting Information). In particular, upon light irradiation, **Indo‐Cy** exhibited the most striking effects on populations of M1 and M2 macrophages, wherein tumor‐promoting M2 macrophages were drastically transformed into tumor‐inhibiting M1 macrophages.

In this respect, **Indo‐Cy** has been demonstrated to enable effective reprogramming of the immunosuppressive microenvironment by means of PDT‐stimulated pyroptosis and **Indo**‐mediated COX‐2 inhibition, leading to amplification of the body's antitumor immune responses. Overall, the developed potent antitumor agent could effectively inhibit the growth of primary and distant tumors. Moreover, the histological analysis of each group (Figure S24, Supporting Information) revealed no obvious morphological or histopathological abnormalities in other organs, suggesting that the proposed therapeutics have negligible dark toxicity. In a pivotal analysis pertaining to the biocompatibility of **Indo‐Cy**, an extensive hematological evaluation was undertaken, encompassing a spectrum of parameters: white blood cell (WBC) count, red blood cell (RBC) count, hemoglobin (HGB) concentration, hematocrit (HCT) level, mean corpuscular volume (MCV), mean corpuscular hemoglobin (MCH), mean corpuscular hemoglobin concentration (MCHC), and platelet (PLT) count. Furthermore, a battery of hepatic function indices was assessed, including total protein (TP), alanine aminotransferase (ALT), aspartate aminotransferase (AST), albumin (ALB), globulin (GLOB), and the albumin‐to‐globulin ratio (A/G). Renal function was equally subject to rigorous examination through the determination of blood urea nitrogen (BUN) and creatinine (CRE) levels. The data elucidated in Figure S25 (Supporting Information) reveal no significant deviations in the aforementioned hematological and biochemical parameters in mice subjected to **Indo‐Cy** administration over a seven‐day interval relative to the untreated control cohort. This observation is indicative of the benign biosafety profile of **Indo‐Cy**, suggesting an absence of untoward systemic effects, thereby underscoring its potential as a biocompatible agent in therapeutic applications.

## 3 Conclusion 

In summary, with the aim of overcoming the inherent hypoxia and immunosuppressive microenvironment of tumors and thus maximizing the antitumor immunotherapy effect of PDT‐activated pyroptosis, a one stone‐three birds effect was engineered with the photon‐induced pyroptosis dye **Indo‐Cy**, which consists of a COX‐2‐inhibiting component and a PDT component of **Cy**. To our surprise, the originally oxygen‐dependent Type II photosensitizer of **Cy** was successfully converted into an anti‐hypoxia Type I photosensitizer. Hence, this conjugate could drastically amplify its PDT potency in hypoxic tumors. Moreover, the additional **Indo** components promoted targeted **Indo‐Cy** accumulation in the ER. Therefore, amplified and intensified PDT could stimulate significant pyroptosis in affected tumor cells. Furthermore, the **Indo** component could also inhibit immunosuppressive COX‐2 and its downstream PGE_2_ synthesis, leading to the conversion of the “cold” immunosuppressive tumor microenvironment into an immunostimulatory “hot” tumor microenvironment. Therefore, our proposed multifaceted PDT conjugate has been demonstrated to not only exert amplified effects on suppressing the growth of primary tumors but also to significantly inhibit the growth of distant tumors. Pertaining to the molecular design, it is particularly worth emphasizing that the linkage of the **Indo** moiety not only causes the molecule to target COX‐2 inhibition but also directly upgrades the Type II photosensitizing parent, **Cy**, to a highly efficient Type I anti‐hypoxia photosensitizer and elucidates for the first time the unique Type I photodynamic mechanism of **Indo‐Cy** under the excited‐state double radical configuration in depth through theoretical calculations. This important information provides a new strategy for the design of pharmacologically active type I photosensitizers.

## 4 Ethical Statement

All the animal experiments involved in this study were conducted in accordance with the Guide for the Care and Use of Laboratory Animals published by the US National Institutes of Health (8th edition, 2011), and approved by the local research ethics review board of the Animal Ethics Commolittee of Dalian University of Technology (Ethics Approval Number: DUTSCE240305‐08).

## Conflict of Interest

The authors declare no conflict of interest.

## Supporting information



Supporting Information

## Data Availability

The data that support the findings of this study are available in the supplementary material of this article.
