# Supplementary material for: A One Stone Three Birds Paradigm of Photon‐Driven Pyroptosis Dye for Amplifying Tumor Immunotherapy
Source: Adv Sci (Weinh). 2025 Jan 13;12(9):2409007. doi: 10.1002/advs.202409007 (PMC11884606; doi:10.1002/advs.202409007)
Supplement: Supplementary file 1 — Supporting Information [file ADVS-12-2409007-s001.docx]

# Supporting Information

**A One Stone Three Birds Paradigm of Photon-induced Pyroptosis Dye for Amplifying Tumor Immunotherapy**

Shuang Zeng^†[a][b]^, Chen Chen^†[c]^, Dan Yu^†[d]^, Maojun Jiang^[e]^, Xin Li^[e]^, Xiaosheng Liu^[b]^, Zhihan Guo^[b]^, Yifu Hao^[b]^, Danhong Zhou^[a]^, Heejeong Kim^[f]^, Heemin Kang^[g]^, Jingyun Wang*^[a][b]^, Qixian Chen*^[h]^, Haidong Li*^[a][b]^, Xiaojun Peng^[a]^, and Juyoung Yoon*^[f]^

[a] S. Zeng, D. Zhou, Prof. J. Wang, Prof. H. Li and Prof. X. Peng

State Key Laboratory of Fine Chemicals, Dalian University of Technology, 2 Linggong Road, Hi-tech Zone, Dalian 116024, China

*E-mail: wangjingyun67@dlut.edu.cn; lihd@dlut.edu.cn

[b] S. Zeng, X. Liu, Z. Guo, Y. Hao, Prof. J. Wang and Prof. H. Li

School of Bioengineering, Dalian University of Technology, 2 Linggong Road, Hi-tech Zone, Dalian 116024, China

[c] C. Chen

Shanghai Institute of Materia Medica, Chinese Academy of Sciences, Shanghai 201203, China

[d] D. Yu

Shanghai Changzheng Hospital, Naval Medical University, Shanghai 20000, China.

[e] M. Jiang and X. Li

School of Chemistry, Dalian University of Technology, Dalian 116024, China

[f] H. Kim and Prof. J. Yoon

Department of Chemistry and Nanoscience, Ewha Womans University, Seoul 03760, Korea.

*E-mail: jyoon@ewha.ac.kr

[g] Prof. H. Kang

Department of Materials Science and Engineering, Korea University, Seoul 02841, Korea.

[h] Prof. Q. Chen

Innovation Center of Yangtze River Delta, Zhejiang University, Jiaxing, 314100, China

*E-mail: plasmid@zju.edu.cn

^†^These authors contributed equally to this work and should be considered co-first authors.

**1. Materials**

All general chemicals for fluorescence detection and organic synthesis including 1,3-diphenylisobenzofuran (DPBF), Dihydrorhodamine 123 (DHR 123), Hydroxyphenyl fluorescein (HPF), 2,7-dichlorodihydrofluorescein diacetate (DCFH), and Dihydroethidium (DHE) were purchased from Shanghai Maokang Biotechnology Co., Ltd. Calcium ion fluorescent probe (Fluo-3 AM) and mitochondrial membrane potential assay kit with JC-1 were obtained from Beyotime Biotechnology Co., Ltd. Hoechst 33324, MitoTracker Green FM, and LysoTracker Green DND-26 were purchased from Thermo (Invitrogen). ER-Tracker Green (BODIPY^®^ FL Glibenclamide) and MitoSOX Red were purchased from MKBio. FAM FLICA™ Caspase-1 Kit was purchased from ImmunoChemistry Technologies (Davis, CA, USA). The anti-COX-2 antibody and anti-GSDMD antibody were purchased from Abcam (Cambridge, MA, USA). The anti-Caspase-1 antibody was procured from Adipogen AG (Liestal, Switzerland). The anti-VEGF antibody was procured from Santa Cruz Biotechnology (Dallas, TX, USA). The anti-HIF-1α antibody was procured from Cell Signaling Technology (Boston, MA, USA). The anti-β-actin was purchased from Proteintech Group Inc. (Chicago, USA). Milli-Q water was supplied by the Milli-Q Plus System (Millipore Corporation, Bedford, USA). All other solvents and reagents used in this study were of the analytical grade.

**2. Instrumentation**

^1^H-NMR and ^13^C-NMR spectra of all compounds were performed with Bruker Avance Ⅲ 400 spectrometer. Absorption and emission spectra for **Cy** and **Indo-Cy** were detected with a Lambda 35 UV-visible spectrophotometer (PerkinElmer) and VAEIAN CARY Eclipse fluorescence spectrophotometer (Serial No. FL0812-M018). Mass spectrometric (MS) data were carried out using LTQ Orbit rap XL instruments. Confocal laser scanning microscope (CLSM) images were performed on Olympus FV3000-IX81 confocal laser scanning microscope. Flow cytometry was analysed by Acoustic Focusing Cytometer (Thermo Fisher). The immolunoreactive bands were determined by ChemiDoc XRS+ system. Small animals’ fluorescence imaging was carried out by NightOWL II LB983 living imaging system.

**3. Synthesis of photosensitizers (Figure S1)**

**3.1 Synthesis of compounds Indo-1 and Cy**

Compounds **Indo-1** and **Cy** were prepared according to the literature procedure.^1,2^

**3.2 Synthesis of Indo-2**

Compound **Indo-1** (100 mg, 0.19 mmol) was dissolved in anhydrous DCM (5 mL), TFA (0.5 mL) was added into the system and stirred at room temperature for overnight, the solvent was removed under reduced pressure to get **Indo-2** (80 mg). The TFA salt of **Indo-2** appeared as a white solid and was used directly in next step. MS (ESI, *m/z*): [M+H]^+^ calcd for C_23_H_27_ClN_3_O_3_, 428.17; found, 428.29; [M+Na]^+^ calcd for C_23_H_26_ClN_3_NaO_3_, 450.16; found, 450.28.

**3.3 Synthesis of Indo-Cy**

Compound **Cy** (100 mg, 0.13 mmol) was dissolved in anhydrous DMF (5 mL), HATU (99 mg, 0.26 mmol) and DIEA (48 μL, 0.26 mmol) was added into the system and stirred for 35 min, and then **Indo-2** (60 mg, 0.14 mmol) was added into the system, after stirring at room temperature for overnight, the reaction mixture was directly purified by flash column chromatography on silica gel using MeOH/CH_2_Cl_2_ (v/v, 1/20) to get purple solid **Indo-Cy** (80 mg, 52%). ^1^H NMR (400 MHz, CDCl_3_, δ): 8.71 (s, 1H), 8.38 (d, *J* = 5.1, 2H), 8.28 (d, *J* = 8.7, 1H), 8.12 (d, *J* = 14.8, 1H), 8.03 (s, 1H), 8.01 (s, 1H), 7.99 (s, 1H), 7.80 (d, *J* = 8.1, 2H), 7.68 (s, 1H), 7.66 (s, 2H), 7.64 (s, 1H), 7.61 (s, 1H), 7.58 (d, *J* = 8.7, 1H), 7.52 (t, *J* = 7.4, 1H), 7.39 (d, *J* = 7.9, 2H), 7.08 (s, 1H), 6.92 (d, *J* = 5.7, 1H), 6.89 (d, *J* = 11.0, 1H), 6.66 (d, *J* = 9.0, 1H), 5.77 (d, *J* = 13.0, 2H), 3.70 (s, 3H), 3.46 (s, 2H), 3.20 (d, *J* = 5.8, 2H), 3.05 (d, *J* = 5.7, 2H), 2.20 (s, 3H), 2.01 (s, 6H), 1.44 (s, 4H). ^13^C NMR (126 MHz, DMSO-d_6_, δ) 177.08, 172.37, 169.67, 168.30, 166.15, 155.98, 149.29, 139.88, 138.84, 138.01, 135.52, 134.75, 134.72, 134.05, 131.94, 131.60, 131.36, 130.72, 130.37, 129.49, 128.34, 127.89, 126.94, 125.51, 123.64, 122.72, 114.99, 114.91, 112.25, 111.69, 102.30, 98.85, 55.85, 54.06, 51.65, 42.31, 38.93, 31.65, 27.30, 27.12, 27.02, 18.56 (s), 17.21 (s), 13.84 (s), 12.97. HRMS (ESI, *m/z*): [M-Br]^+^ calcd for C_53_H_48_ClI_2_N_4_O_5_, 1109.1398; found, 1109.1418.

**4. ROS generating ability test**

**Total ROS:** The test was carried out referring to the methods reported in the literature.^3^ We first conducted the transformation of DCFH-DA to DCFH. The activated DCFH-DA solution (DCFH, 4×10^-5^ M) was added into the sample solution (DMSO) containing **Cy** or **Indo-Cy** (10 μM). Upon irradiation with 580 nm laser irradiation (5 mW/cm^2^) for different time intervals. The fluorescence of DCF at 525 nm was measured in a fluorescence spectrophotometer at the excitation of 488 nm. In addition, DCFH alone was tested under the same experimental procedures as the control.

**Singlet oxygen (^1^O_2_):** To bring the absorbance at 415 nm close to 1.0, 1,3-diphenylisobenzofuran (DPBF) was added to a sample solution (DCM) containing **Cy** or **Indo-Cy** (10 μM) as a singlet oxygen indicator. The absorption spectra of DPBF were then measured after the solution was treated with 580 nm laser irradiation (10 mW/cm^2^) for different intervals. The DPBF was then evaluated independently using the same experimental procedures as the control.

**Superoxide radical (O_2_^•-^):** Dihydrorhodamine 123 (DHR123) was added to a sample solution (PBS) containing **Cy** or **Indo-Cy** (10 μM) as a superoxide radical indicator. The fluorescence spectra of DHR123 were then measured after the mixed solution was exposed to 580 nm laser irradiation (10 mW/cm^2^) at various intervals. DHR123 was evaluated independently using the same experimental procedures as the control.

**Hydroxyl radical (•OH):** HPF was added to a sample solution (PBS) containing **Cy** or **Indo-Cy** (10 μM) as an indicator for hydroxyl radicals. The fluorescence spectra of HPF were then measured after the mixed solution was exposed to 580 nm laser irradiation (5 mW/cm^2^) for varying intervals. HPF was evaluated independently using the same experimental procedures as the control.

**5. Determination of singlet oxygen quantum yield (*ɸ_∆_*)**

The Rose Bengal (RB, 10 μM) was tested under the same experimental procedures as the reference. At last, the singlet oxygen quantum yield was calculated with the following equation:

$$ɸ_{\Delta}=ɸ_{RB}\times(K_{Ps}\times F_{RB})/(K_{RB}\times F_{Ps})$$

ɸ_∆_ represents the singlet oxygen quantum yield of the tested photosensitizer; ɸ_RB_ represents the singlet oxygen quantum yield of RB and the value is 0.76; Ps represents thetested photosensitizer; k represents the slope of the decrease of the absorbance at 415 nm of DPBF with the addition of irradiation time; F is the correction factor which is calculated by the following equation:

$$F=1-{10}^{-OD}$$

OD represents the absorbance of the mixture at 580 nm.

**6. Cell culture**

The cancer cells (4T1, MCF7 and HeLa) were acquired from the Institute of Basic Medical Sciences of the Chinese Academy of Science. DMEM high glucose medium was used to cultivate MCF7 and HeLa cells, while RPMI-1640 was used to cultivate 4T1 cells. These cancer cells were cultured in the above medium containing 10% fetal bovine serum and 1% antibiotics (penicillin/streptomycin; 100 U mL^−1^) at 37 °C in a 5% CO_2_ atmosphere.

**7. Molecular theory calculations**

Geometry optimizations in the ground and excited states were carried out using Density functional theory (DFT) and time-dependent DFT (TD-DFT) method with B3LYP functional,^4^ respectively. The SDD basis set on I atom and 6-31g(d) basis set on all other atoms were used.^5^ The excitation calculations were performed with TD-DFT by using the TZVP basis set for all other atoms except for I atom.^6^ Solvent effect was accounted for according to the experimental conditions by using the polarized continuum model (PCM) with water as the solvent.^7^ All calculations were carried out with the Gaussian 16 package. The charge density difference was calculated using the Multiwfn program.^8^

1. **Molecular dynamics simulation**

A 200 ns molecular dynamics simulation was conducted for the COX2/**Indo-Cy** complex using Desmond 2020. The OPLS3e all-atom force field was employed to derive force field parameters for the protein system. The COX2/**Indo-Cy** complex was submerged in a TIP3 water molecule-containing box. To neutralize the charges of the simulation systems, an appropriate number of Na^+^ and Cl^-^ counter ions were added to the solvated systems (protein, inhibitor, and water). Hydrogen atom positions were constrained using the SHAKE algorithm. During the whole MD simulation, particle mesh Ewald (PME) was applied to treat the long-range electrostatic interactions with a periodic boundary condition. The system was equilibrated using the NPT ensemble at 300 K and 1.0 bar. Temperature-pressure coupling was performed using the Berendsen coupling algorithm. The MD simulations involved a 200 ns run with a time step of 1.2 fs, and trajectory recording occurred every 10 ps, resulting in a total of 20,000 frames. The root mean square deviation (RMSD) of the main chain atoms was computed and visually assessed to comprehend the characteristics of COX2/**Indo-Cy** interactions. Additionally, the root mean square fluctuation (RMSF) of each residue was calculated to identify significant conformational changes between the initial and dynamic states.

**9. Enzyme activity inhibition assay of COX-2 *in vitro***

Prepare the corresponding detection solution according to the instructions of COX-2 Inhibitor Screening Kit (Beyotime, China), and the positive inhibitor is indomethacin solution. Add the test sample and working solution to the 96-well plates in sequence, and incubate at 37°C for 1 hour. Subsequently, COX-2 probes were added to each well and incubated at 37°C for 5 minutes in the dark and then measure fluorescence intensity with a microplate reader at Ex/Em =560/590nm. Calculate the average Relative Fluorescence Unit (RFU) for each sample and control wells and the inhibition rate was calculated using the following formula:

$$Inhibition rate (\%)=\frac{{{RFU}_{100\% Enzyme Activity Control}-RFU}_{Sample}}{{{RFU}_{100\% Enzyme Activity Control}-RFU}_{Blank}}\times100\%$$

**10. Intracellular tracking**

This study employed both MCF-7 and 4T1 cells. The cells were incubated with fresh medium containing **Indo-Cy** (5 μM) for 15 min before being washed with PBS and stained with 1 μM commercialized dyes (Hoechst 33324, MitoTracker Green, LysoTracker Green, and ER-Tracker Green) for 15 min. After washing the samples with PBS, the subcellular localization of **Indo-Cy** was examined using CLSM imaging. Conditions: excitation wavelength: 400 nm for Hoechst 33324, 488 nm for MitoTracker Green, LysoTracker Green, ER-Tracker Green and 580 nm for **Indo-Cy**; emission filter: 450–480 nm for Hoechst 33324, 510–530 nm for MitoTracker Green, LysoTracker Green, ER-Tracker Green and 600–700 nm for **Indo-Cy**. Following the collection of fluorescence images, they were analyzed using the ImageJ software.

**11. Intracellular ROS imaging**

The production of O_2_^•–^, •OH, and total ROS in 4T1 cells was demonstrated using DHE, HPF, and DCFH-DA, respectively. 4T1 cells were plated on 35 mm confocal dishes, incubated with DCFH-DA (10 μM) for 30 min, and then stained with **Indo-Cy** (10 μM) for 30 min, using DCFH-DA as an example. The light group was exposed to 580-nm LED light (40 mW; 5 min; 12 J/cm^2^) following washing with serum-free medium. Subsequently, confocal fluorescence imaging was conducted and the corresponding images were acquired. Conditions: excitation wavelength for DCF was 488 nm, and the emission wavelength ranged from 500–530 nm. For other ROS evaluations, the same procedure as for DCFH-DA measurement was followed. In addition, to simulate a hypoxic tumor environment, cells were cultured in an incubator chamber (MIC-101, Billups–Rothenberg) at 37 ℃ in a humidified, 2% O_2_ and 5% CO_2_ atmosphere while the oxygen content of the chamber was monitored using an oxygen detector (Nuvair, O2 Qucikstick). Other operations were identical to those conducted in a normoxic environment.

**12. *In vitro* cytotoxicity assays**

4T1 cells were plated at 1 × 10^5^ cells per well in a 96-well cell culture plate. This was followed by 24 h of incubation at 37 °C in either a normoxic (21% O_2_) or hypoxic (2% O_2_) atmosphere. The cells were then incubated for 1 h with varying concentrations of **Indo-Cy** under normoxic (21% O_2_) or hypoxic (2% O_2_) conditions. After washing the cells with 100 μL of fresh medium, they were exposed to a 580 nm LED lamp with a power density of 40 mW/cm^2^ for 5 min and allowed to continue growing for 24 h. Subsequently, fresh medium (100 μL) and MTT (10 μL, 5 mg/mL) were added to each well and the cells were incubated for an additional 4 h at 37 °C. Finally, the absorbance of 570 nm was then measured using a Bio-Rad microplate reader, and the cell viability was computed. In this experiment, no light exposure was used to measure the dark toxicity of different PS; all other procedures were identical.

**13. Live/dead cell co-staining**

4T1 cells were plated onto 6-well plate and incubated with 5 μM **Indo-Cy** for 1h, the light group was irradiated with 580 nm LED light (12 J/cm^2^ or 24 J/cm^2^). Cultures were continued for an additional 4 h at 37 °C in 5% CO_2_, then stained with Calcein-AM/PI Double Stain Kit according to the instruction manual. After that, the samples were imaged by CLSM, the excitation wavelength was 488 nm for Calcein-AM and PI, the emission wavelength was 500-530 nm for Calcein-AM and 650-750 nm for PI.

**14. Annexin V-FITC & PI staining**

4T1 cells were incubated with 5 μM **Indo-Cy** for 1h, then washed with DMEM and irradiated with 580 nm LED light (40 mW, 10 min, 24 J/cm^2^) and incubated for an additional 1h at 37°C. Then stained with Annexin V-FITC/propidium iodide (PI) Stain Kit according to the instruction manual. The fluorescence images collected by CLSM, the fluorescence of Annexin V-FITC was excitated at 488 nm and collected at 500-540 nm. The fluorescence of PI was excitated at 488 nm and collected at 650-750 nm.

**15.** **Intracellular calcium ion imaging**

Fluo-3 AM was used to detect photoinduced calcium ion release on the ER mediated by **Indo-Cy**. 4T1 cells were incubated for 0.5 h with 10 μM **Indo-Cy**, followed by 15 min with 5 μM Fluo-3 AM. Following a PBS wash, cells were exposed to 580 nm LED light for 5 min at 40 mW/cm^2^ with a power density of 40 mW/cm^2^. Following irradiation, fluorescent images were captured at different times. The excitation wavelength was 488 nm and the collected emission wavelength ranged between 510 and 540 nm.

**16.** **Mitochondrial membrane potential assay**

JC-1 was used as the mitochondrial membrane potential indicator. Briefly, 4T1 cells were seeded onto 35 mm confocal dishes for 24 h, then cells were treated with following different treatments: group 1, untreated (Control); group 2, incubated with 10 μM CCCP at 37 ℃ for 0.5 h (CCCP, positive control); group 3, incubated with 10 μM **Indo-Cy** at 37 ℃ for 0.5 h (**Indo-Cy**); group 4, incubated with 10 μM **Indo-Cy** for 0.5 h at 37℃ and irradiated with 580 nm LED light for 5 min at a power density of 40 mW/cm^2^, then continue incubation for 1 h. (**Indo-Cy**+L). Before imaging experiments, all cells were stained with JC-1 for another 20 min. The excitation wavelength of JC-1 monomers was 488 nm, and capture emission region was 500-520 nm; the excitation wavelength of JC-1 aggregates was 560 nm, and capture emission region was 570-590 nm.

**17.** **Mitochondrial superoxide fluorescence imaging**

MitoSOX Red was used to detect photoinduced mitochondrial superoxide mediated by **Indo-Cy**. 4T1 cells were incubated with 10 μM **Indo-Cy** for 1 h, washed with PBS, and then exposed to 580 nm LED light at a power density of 40 mW/cm^2^ for 5 min. The fluorescent images were then captured at various intervals after irradiation and were incubated with 5 μM MitoROS for 10 min before imaging. The excitation wavelength was 488 nm, and the emission collection wavelength ranged between 570 and 600 nm.

**18. CRT and HMGB1 staining in 4T1 cancer cells**

4T1 cells were plated onto 35 mm confocal dishes and incubated with 5 μM **Indo-Cy** for 1h, then the cells were washed and irradiated by 580 nm LED lamp irradiation (40 mW/cm^2^) for 10 min, After 12 h, the cells were washed with pre-cooled 1×PBS, fixed with 4% paraformaldehyde on the ice for 1 min, and further incubated with Alexa Fluor 488 labelled CRT antibody or Alexa Fluor 488 labelled HMGB1 antibody for 1 h. The CRT and HMGB1 expression fluorescence images collected by CLSM with the excitation at 488 nm and signal acquisition in the range from 500 to 540 nm.

**19.** **ATP release assays**

4T1 cells were plated at 1×10^5^ cells per well in a 96-well cell-culture plate, followed by incubation at 37 °C for 24 h. Then the cells were incubated with 5 μM **Indo-Cy** for 1h and washed with 100 μL fresh medium. The light groups were irradiated with 580 nm LED lamp at a power density of 40 mW/cm^2^ for 5 min and continued growing for 4 h. The ATP release were detected by Enhanced ATP Assay Kit (Beyotime).

**20. LDH release assays**

4T1 cells were plated at 1×10^5^ cells per well in a 96-well cell-culture plate, followed by incubation at 37 °C for 24 h. Then the cells were incubated with 5 μM **Indo-Cy** for 1h and washed with 100 μL fresh medium. The light groups were irradiated with 580 nm LED lamp at a power density of 40 mW/cm^2^ for 5 min and continued growing for 12 h. The LDH release were detected according to the instruction manual.

**21. Intracellular** **caspase-1 imaging**

First, 4T1 cells were incubated on the cell culture plate for 24 h and then exposed to different treatments: group 1 untreated (Control); group 2, incubated with 10 μM **Indo** at 37 ℃ for 0.5 h (**Indo**); group 3, incubated with 10 μM **Indo** for 0.5 h at 37℃ and irradiated with 580 nm light for 5 min at a power density of 40 mW/cm^2^ (**Indo** + L); group 4, incubated with 10 μM **Indo-Cy** at 37 ℃ for 0.5 h (**Indo-Cy**); group 35, incubated with 10 μM **Indo-Cy** for 0.5 h at 37℃ and irradiated with 580 nm light for 5 min at a power density of 40 mW/cm^2^ (**Indo-Cy** + L). Using the FAM FLICA™ Caspase-1 Kit, active caspase-1 enzyme was labeled in living cells following various treatments. The excitation wavelength for the green channel was 488 nm, and the emission wavelength ranged from 510 to 540 nm.

**22. Western blotting analysis**

For caspase-1 and GSDMD evaluation, 4T1 cells treated with **Indo-Cy** (10 μM) upon light irradiation or under dark conditions were collected and subjected to standard western blot. Controls consisted of untreated 4T1 cells. The extracted proteins were separated using SDS-PAGE and then transferred to polyvinylidene difluoride (PVDF) membranes. To prevent interference from nonspecific binding, the membranes were blocked with 5% nonfat milk solution and incubated overnight at 4°C with the indicated primary antibodies against caspase-1 and GSDMD. After incubation with the goat-antirabbit IgG-HRP secondary antibody (Thermo Fisher scientific, Oregon, USA) at room temperature for 2 h, the immunoreactive bands were detected using a chemiluminescence (ECL) system (Bio-Rad, CA, USA). For COX-2, HIF-1α and VEGF, 4T1 cells treated with **Indo-Cy** (50 μM) for 24 h and other procedures were consistent with caspase-1 evaluation.

**23. Bone marrow dendritic cell (BMDC) culture and stimulation**

Mouse bone marrow dendritic cells (BMDCs) were isolated from the femur and tibia of female BALB/c mice. Briefly, after the lysis of red blood cells, cells were inoculated into a cell culture dish with 10 mL complete RPMI 1640 medium (20 ng/mL of GM-CSF and 10 ng/mL of IL-4) at 37 °C for 6 days to acquire immature DCs. 4T1 cells were seeded into 6‐well culture plates at a density of 1 × 10^6^ cells/well and incubated at 37 °C. After growing full over the plate, 4T1 cells were treated with 10 μM **Indo-Cy** for 2 h and then washed with 1 mL fresh medium. The light groups were irradiated with 580 nm LED lamp at a power density of 40 mW/cm^2^ for 5 min and continued culturing for 6 h. For no light groups, no light irradiation was applied to this experiment, and all other steps were consistent with the light groups. Subsequently, the supernatants were collected and co-cultured with the immature BMDCs for 24 h. After staining with anti-CD11c-FITC, anti-CD80-APC, and anti-CD86-PerCP-Cy5.5 antibodies, the BMDCs were washed and resuspended in PBS containing 1% FBS for flow cytometry analysis.

**24. *In vitro* wound healing assay**

4T1 cells were seeded into 6‐well culture plates and incubated at 37 °C for 24 h to grow full over plate. After the cells were incubated with different photosensitizers (50 μM), a line in the middle of culture dishes was drawn with a pipette and washed with PBS for 3 times. Cells were incubated for another 24 h before imaging.

**25. Antitumor capacity *in vivo***

The 4–6 week old BALB/c mice were chosen and 4T1 tumors were established by s. c. injecting 2 × 10^6^ 4T1 cells. When the primary tumor volume reached 100 mm^3^, the tumor-bearing mice were randomly divided into four groups (PBS, **Indo**, **Indo-Cy**, **Indo-Cy** + Light, n = 5). The light group were irradiated with 580 nm laser for 20 min at photodensity of 100 mW/cm^2^. The distant tumor were established by s.c. injecting 1 × 10^5^ 4T1 cells. As for PDT, the mice injected with 100 μM (100 μL) different photosensitizers. The tumor volume was calculated by using the formula:

$$\text{v=}\frac{\text{1}}{\text{2}}\text{×a×}\text{b}^{\text{2}}$$

V represents the tumour volume of mice, *a* represents the longest diameter of the tumour region, and *b* represents the diameter in the vertical direction according to a of the tumour region. All the animal experiments involved in this study were conducted in accordance with the Guide for the Care and Use of Laboratory Animals published by the US National Institutes of Health (8th edition, 2011), and approved by the local research ethics review board of the Animal Ethics Commolittee of Dalian University of Technology (Ethics Approval Number: DUTSCE240305-08).

**26. Analysis of immune cells.**

To systematically investigate the *in vivo* antitumor immune responses, primary tumors and distant tumors of mice were harvested. These tissues were made into a single cell suspension according to the specified procedure. For the analysis of T cells, tumor cells were stained with FITC anti-mouse CD3, APC anti-mouse CD8a, and PE/Cyanine7 anti-mouse CD4 antibodies (BioLegend) according to the manufacturer’s protocols. For the analysis of regulatory cells (Tregs), tumor cells were stained with FITC anti-mouse CD3, PE anti-mouse FOXP3 and PE-Cyanine7 anti-mouse CD4 antibodies (BioLegend) according to the manufacturer’s protocols. For the analysis of M1 and M2 cells, tumor cells were stained with PE anti-mouse F4/80, and FITC anti-mouse CD206 antibodies (BioLegend).

**27. Statistical analysis**

All the experiments were performed for three times and the data were presented as means ± standard deviation (S.D.). Statistical analysis was performed according to the one-way analysis of variance (ANOVA) analysis by SPSS software. Differences were considered statistically significant at the following p values: *p < 0.05, **p < 0.01, ***p < 0.001.

**Supporting Figures**

**Figure S1.** Synthetic routes of photosensitizer **Indo-Cy**.


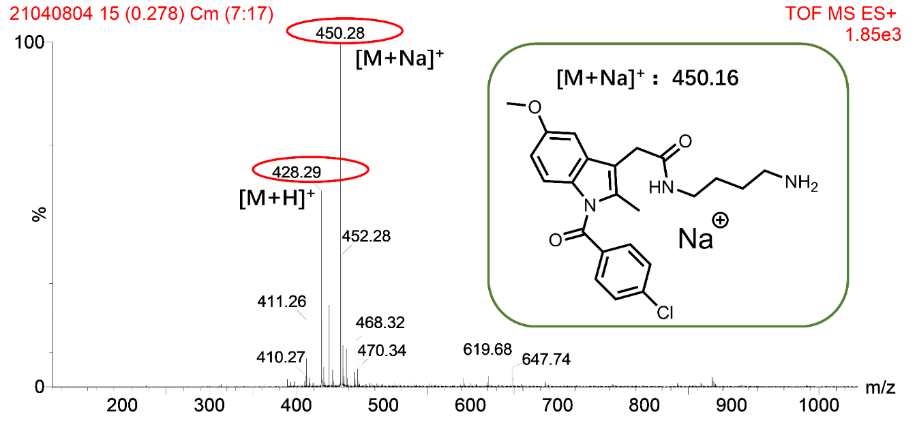


**Figure S2.** MS spectrum of Compound **Indo-2**.


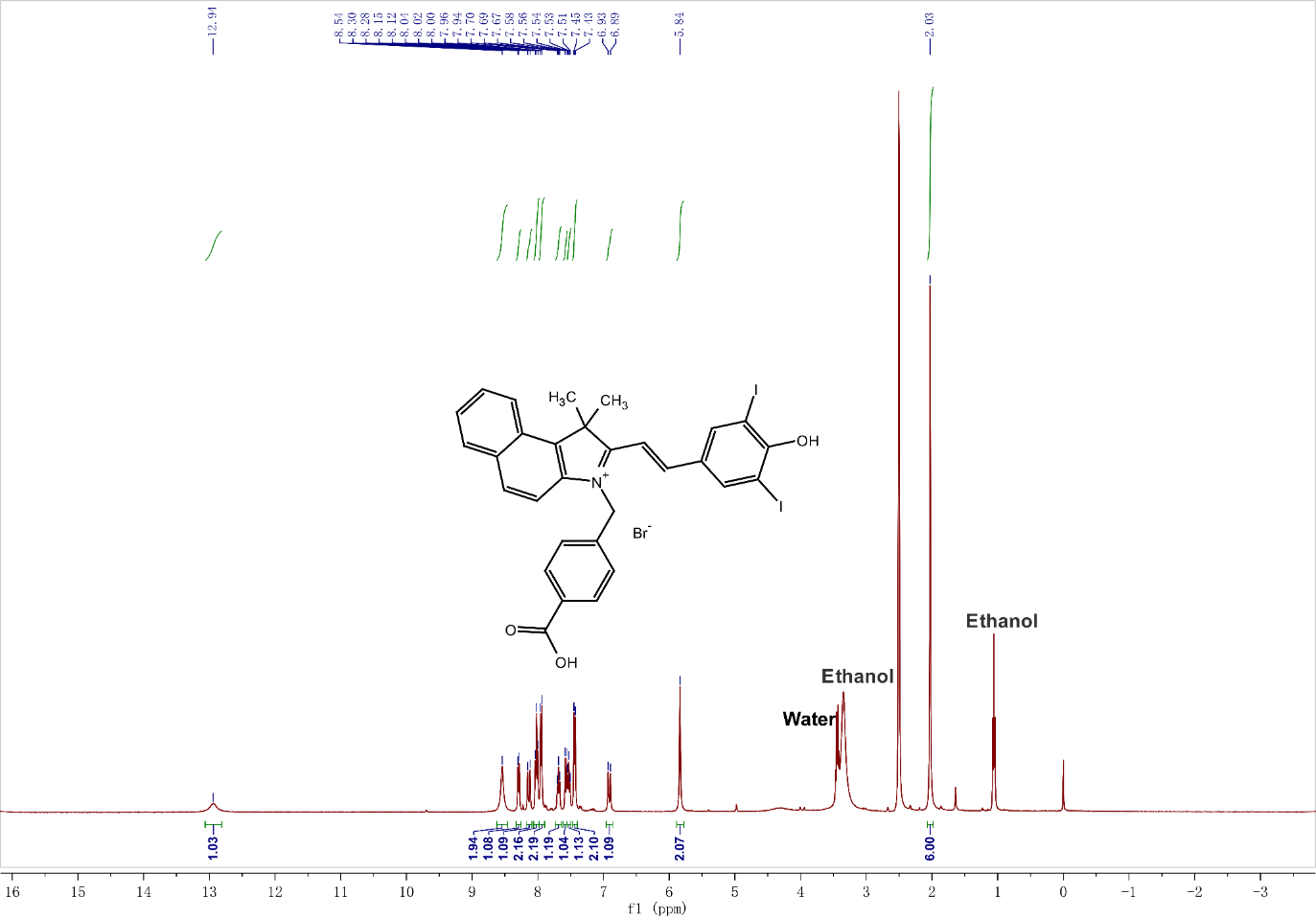


**Figure S3.** ^1^H-NMR spectrum of compound **Cy** in DMSO**-**d_6_.


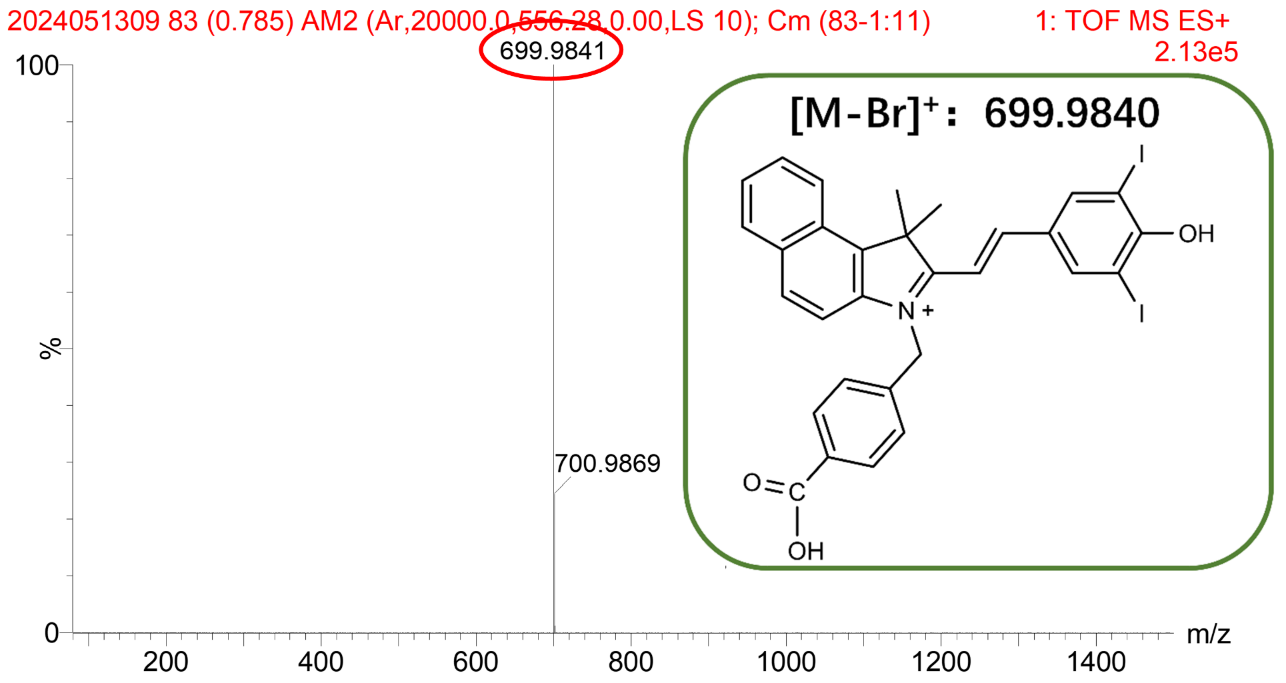


**Figure S4.** HRMS spectrum of compound **Cy**.


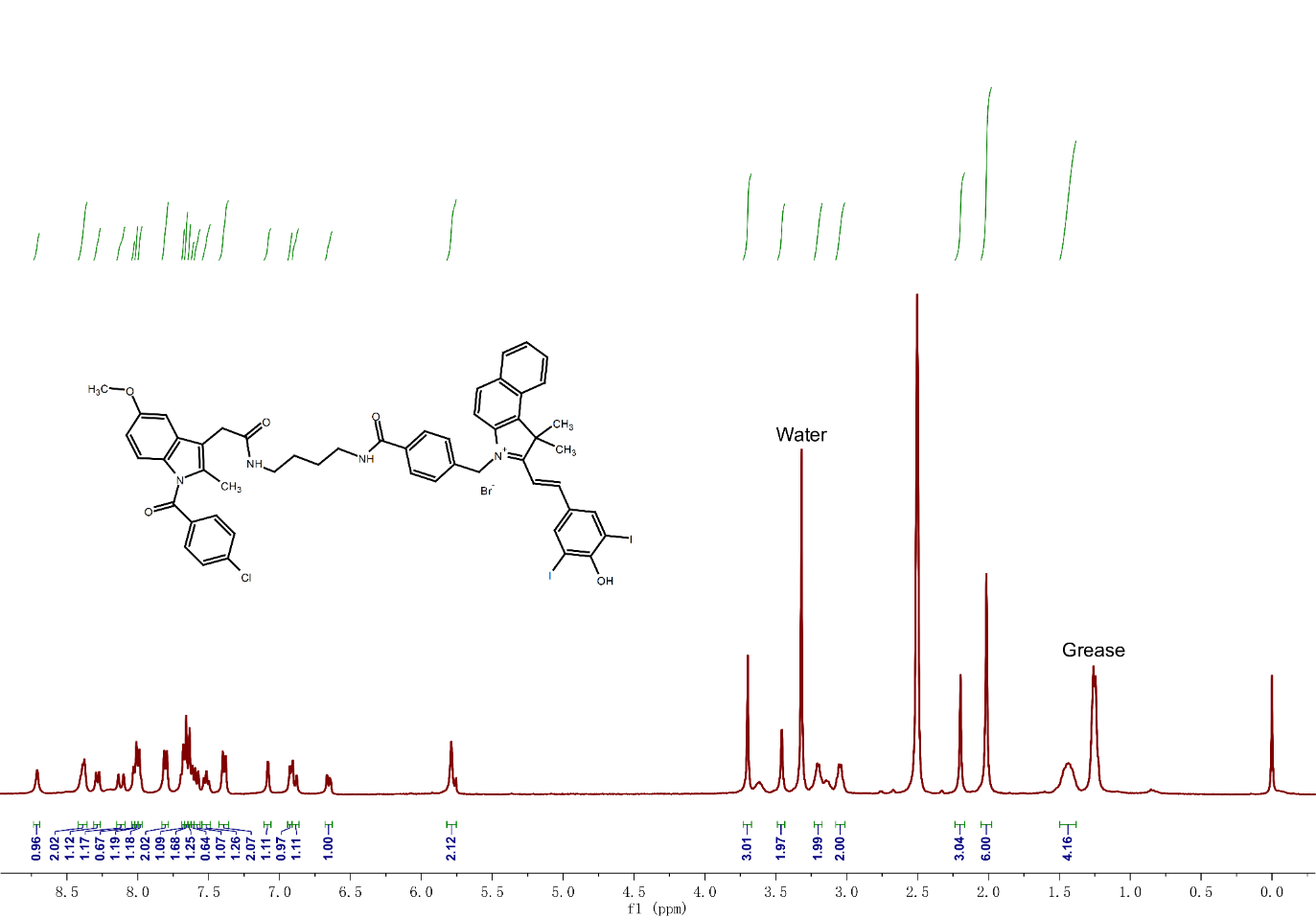


**Figure S5.** ^1^H-NMR spectrum of photosensitizer **Indo-Cy** in CDCl_3_.

**
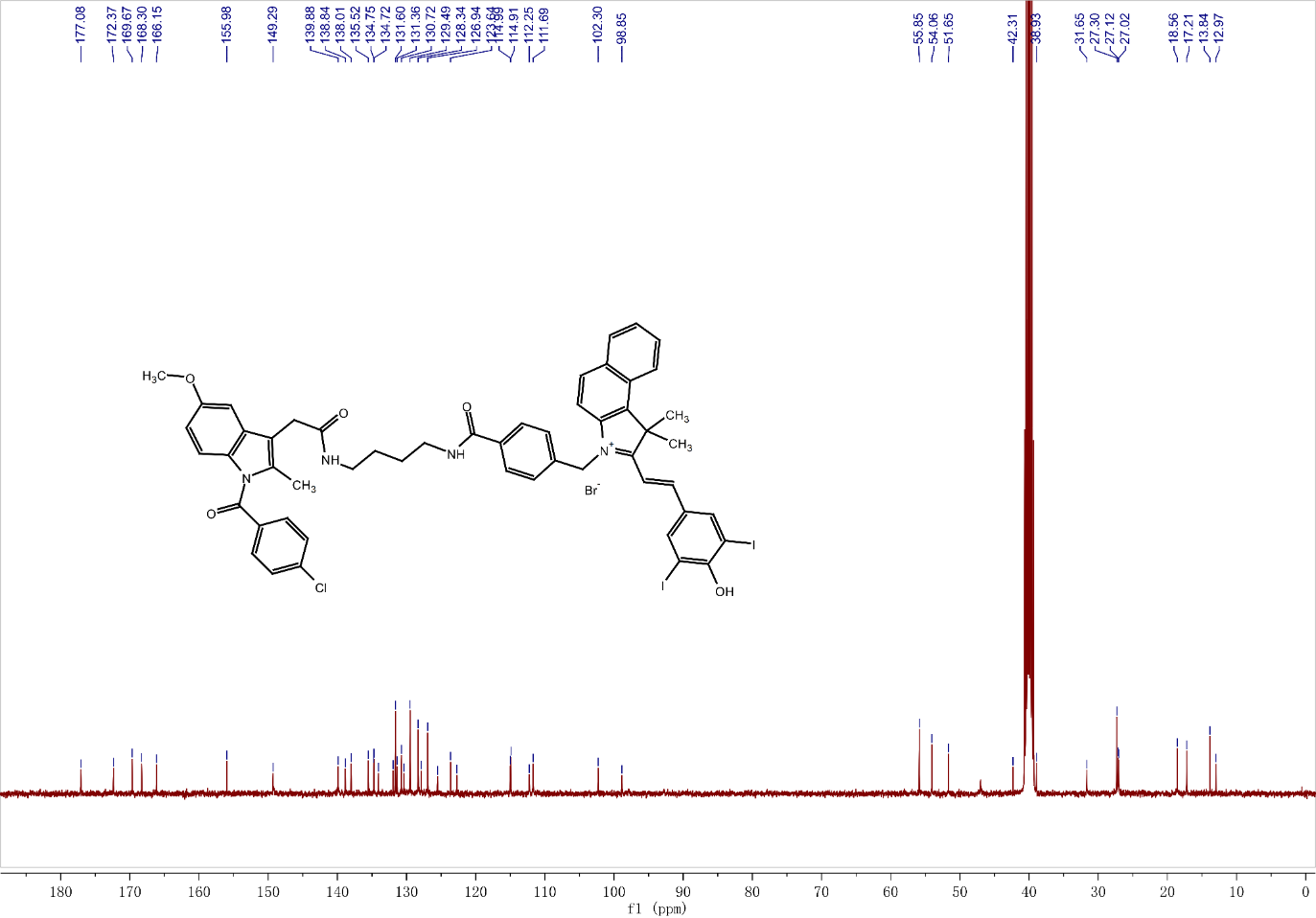
**

**Figure S6.** ^13^C-NMR spectrum of photosensitizer **Indo-Cy** in DMSO**-**d_6_.


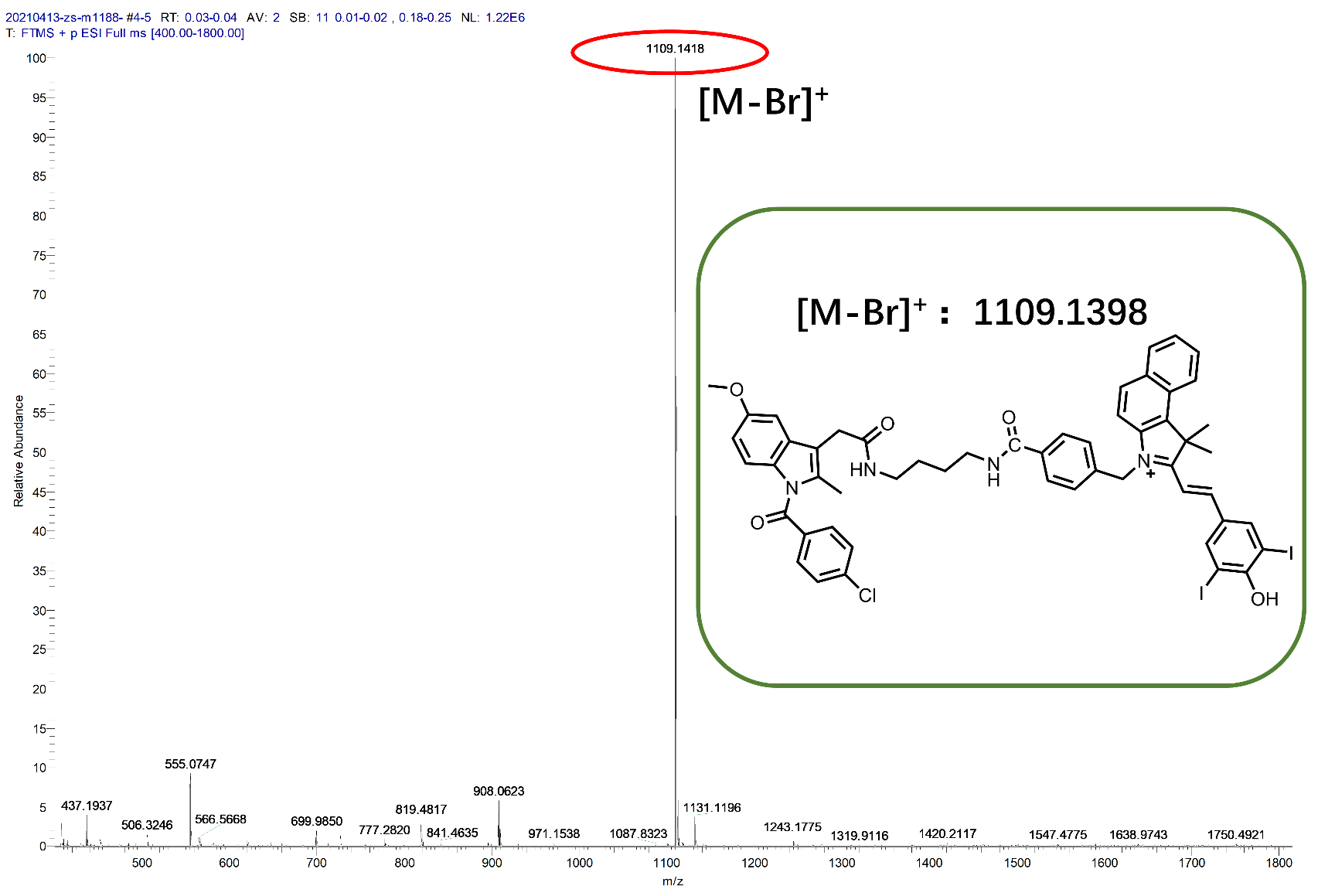


**Figure S7.** HRMS spectrum of Compound **Indo-Cy**.


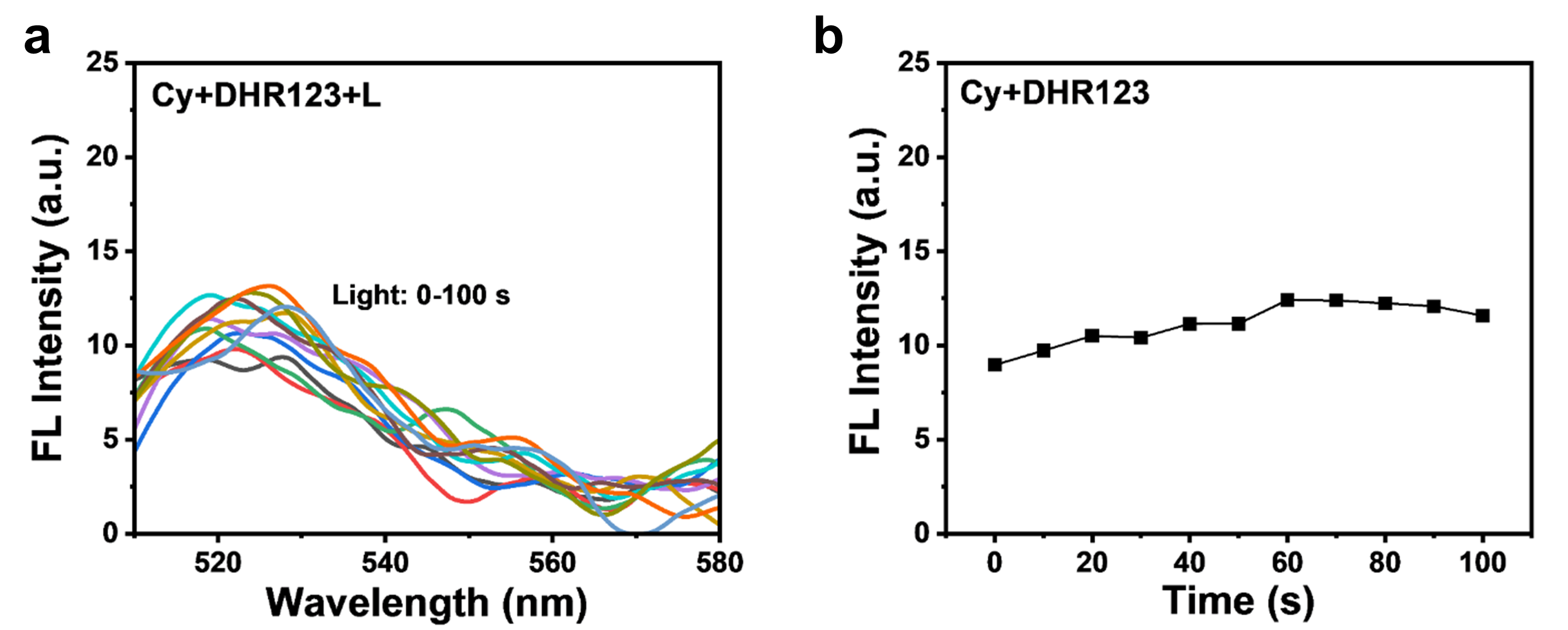


**Figure S8**. Fluorescent spectra of DHR123 (a) and the intensity changes at 525 nm (b) under different irradiation time with **Cy** (10 μM), respectively.


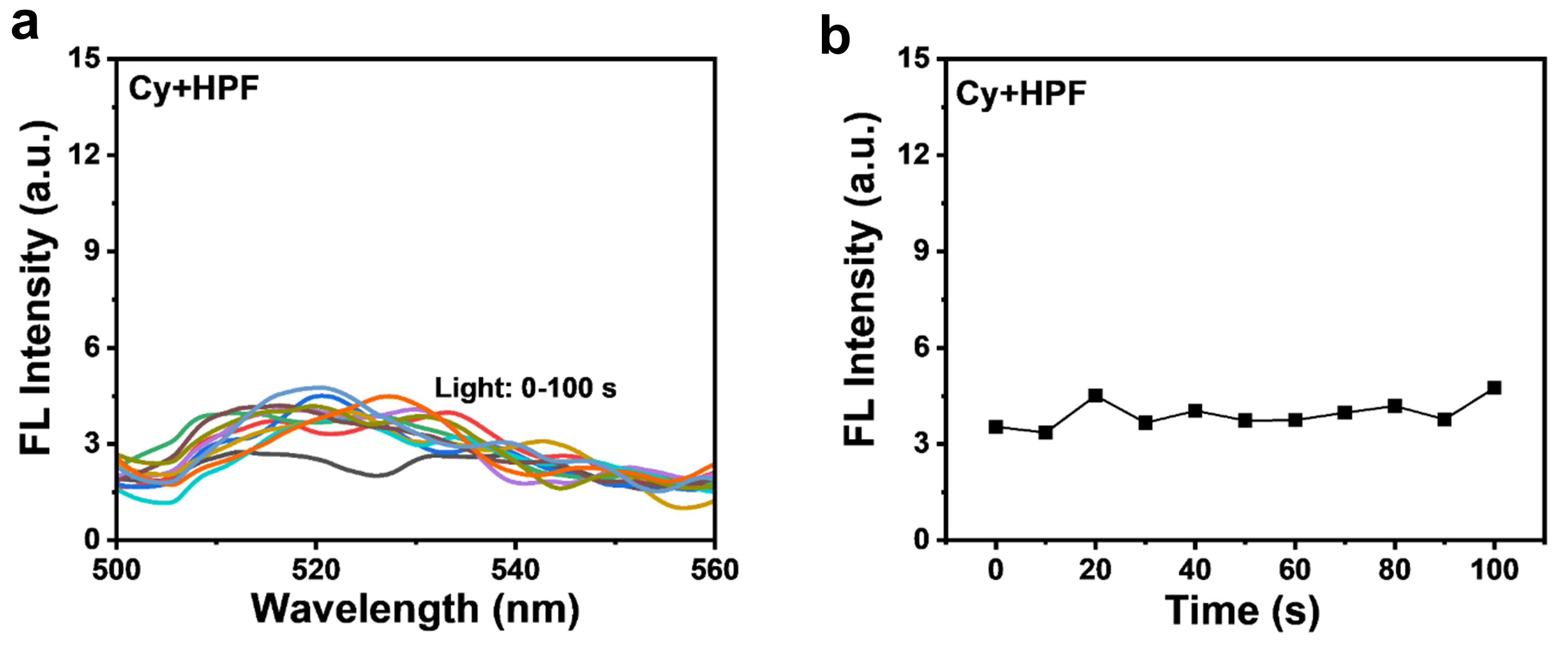


**Figure S9**. Fluorescent spectra of HPF (a) and the intensity changes at 515 nm (b) under different irradiation time with **Cy** (10 μM), respectively.


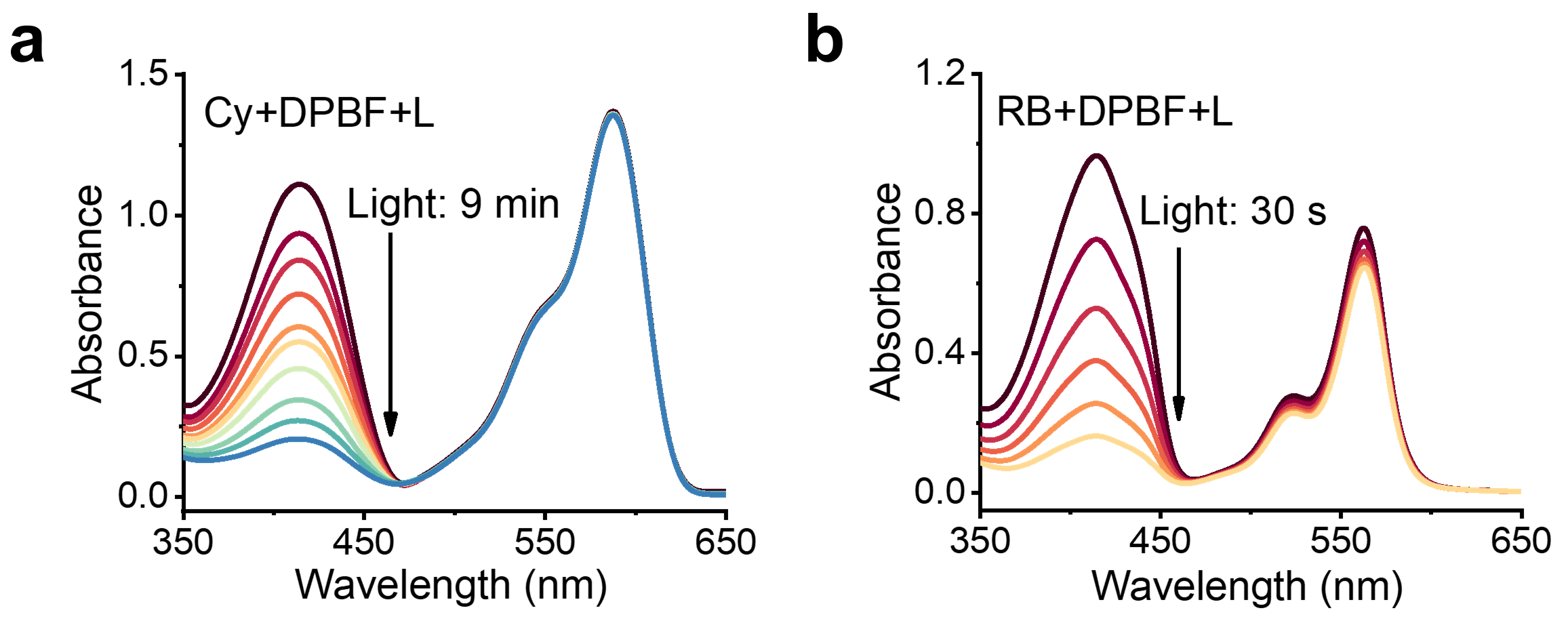


**Figure S10.** Absorption spectra of DPBF containing (a) **Cy** and (b) **RB** (10 μM) as a function of irradiation periods, respectively.


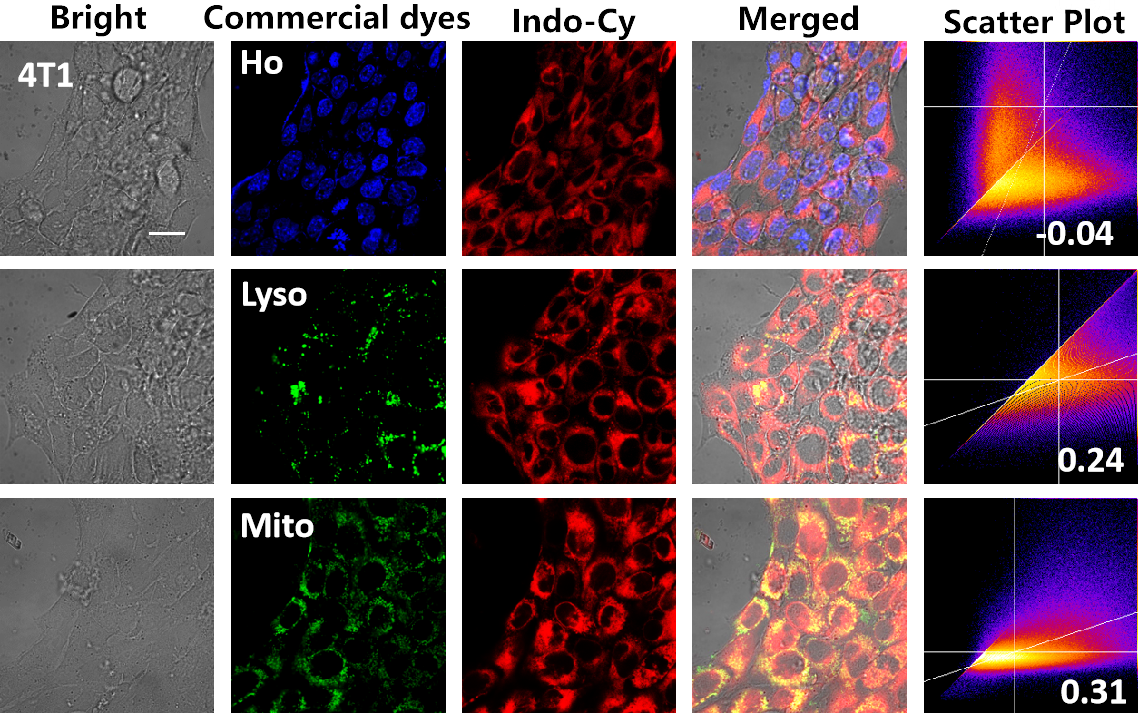


**Figure S11**. The distributions of **Indo-Cy** (red, λ_ex_ = 580 nm, λ_em_ = 600-700 nm) in 4T1 cells by CLSM observation, wherein nucleus, lysosomes and mitochondria were stained by commercial dyes of Hoechst 33324 (λ_ex_ = 400 nm, λ_em_ = 450-480 nm), Mito-Tracker Green or Lyso-Tracker Green (λ_ex_ = 488 nm, λ_em_ = 510-530 nm), respectively. Scale bars: 20 µm.


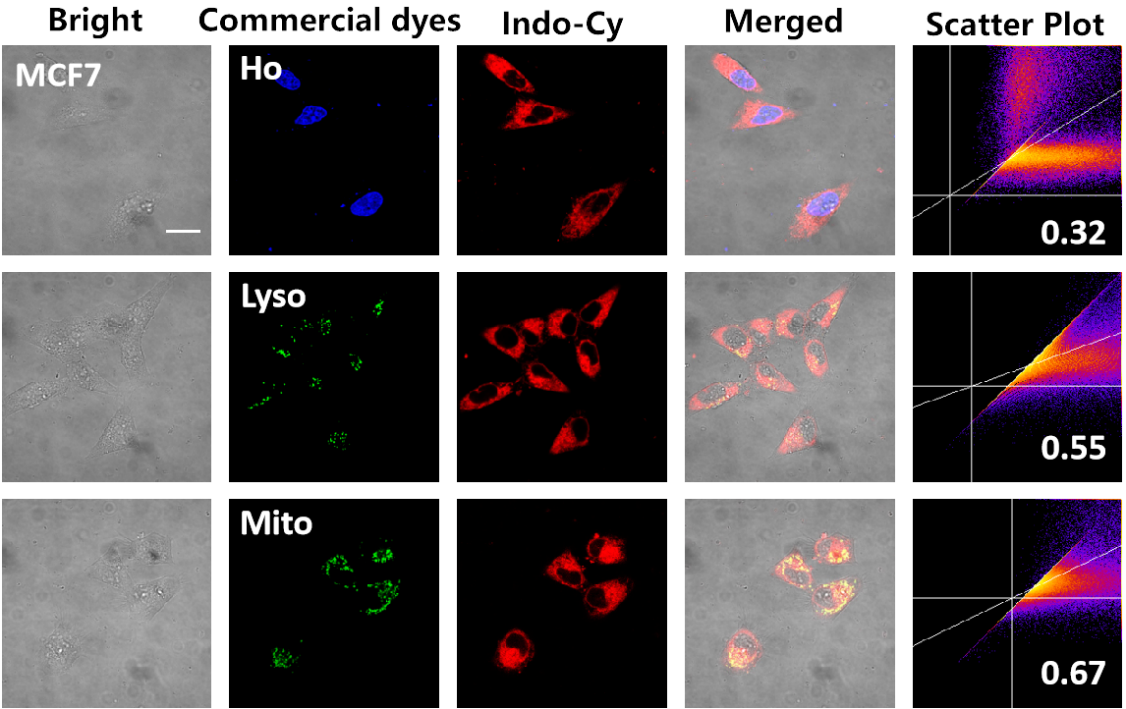


**Figure S12**. The distributions of **Indo-Cy** (red, λ_ex_ = 580 nm, λ_em_ = 600-700 nm) in MCF7 cells by CLSM observation, wherein nucleus, lysosomes and mitochondria were stained by commercial dyes of Hoechst 33324 (λ_ex_ = 400 nm, λ_em_ = 450-480 nm), Mito-Tracker Green or Lyso-Tracker Green (λ_ex_ = 488 nm, λ_em_ = 510-530 nm), respectively. Scale bars: 20 µm.


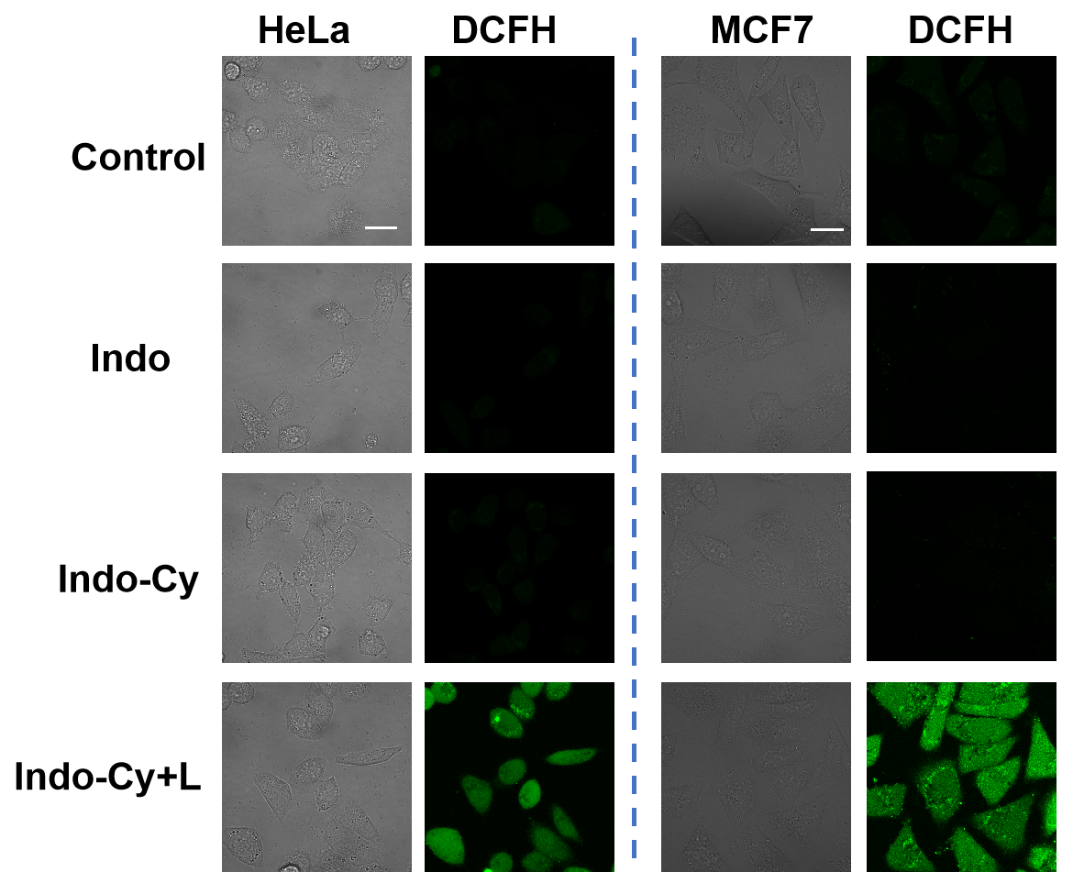


**Figure S13.** ROS production in HeLa and MCF-7 cells treated with Indo-Cy (10 μM) and measured using DCFH-DA as a fluorescence indicator (green, λ_ex_ = 488 nm, λ_em_ = 510–530 nm), respectively. The light group was exposed to 580-nm LED light (40 mW; 5 min; 12 J/cm^2^). Scale bars: 20 μm.


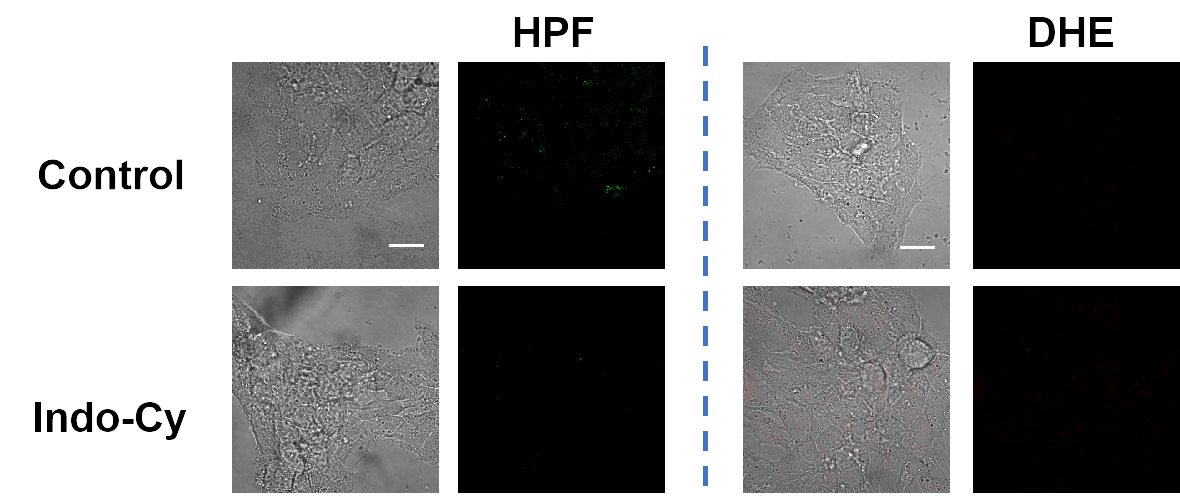


**Figure S14.** ROS production in 4T1 cells using DHE and HPF as the O_2_^•-^ and •OH fluorescence indicators, respectively.


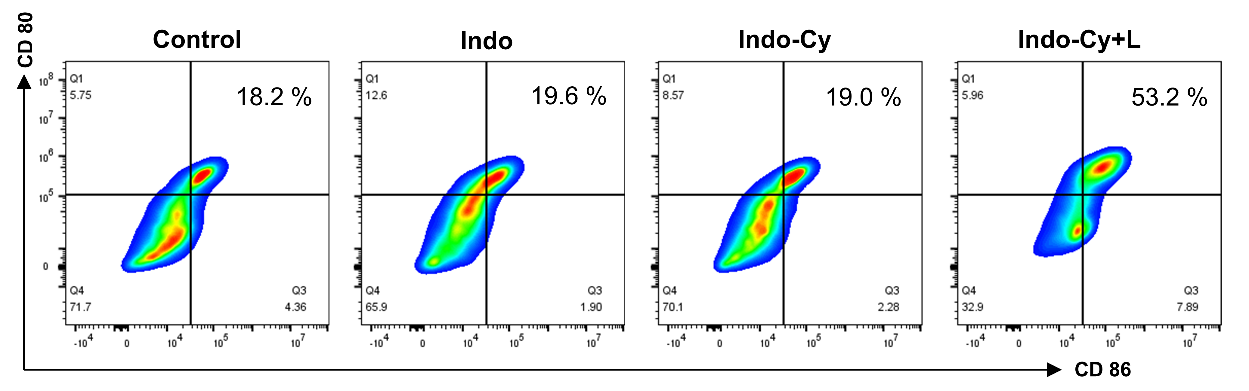


**Figure S15.** Quantitative assessment of CD80 and CD86 expression on dendritic cells by Flow Cytometry.


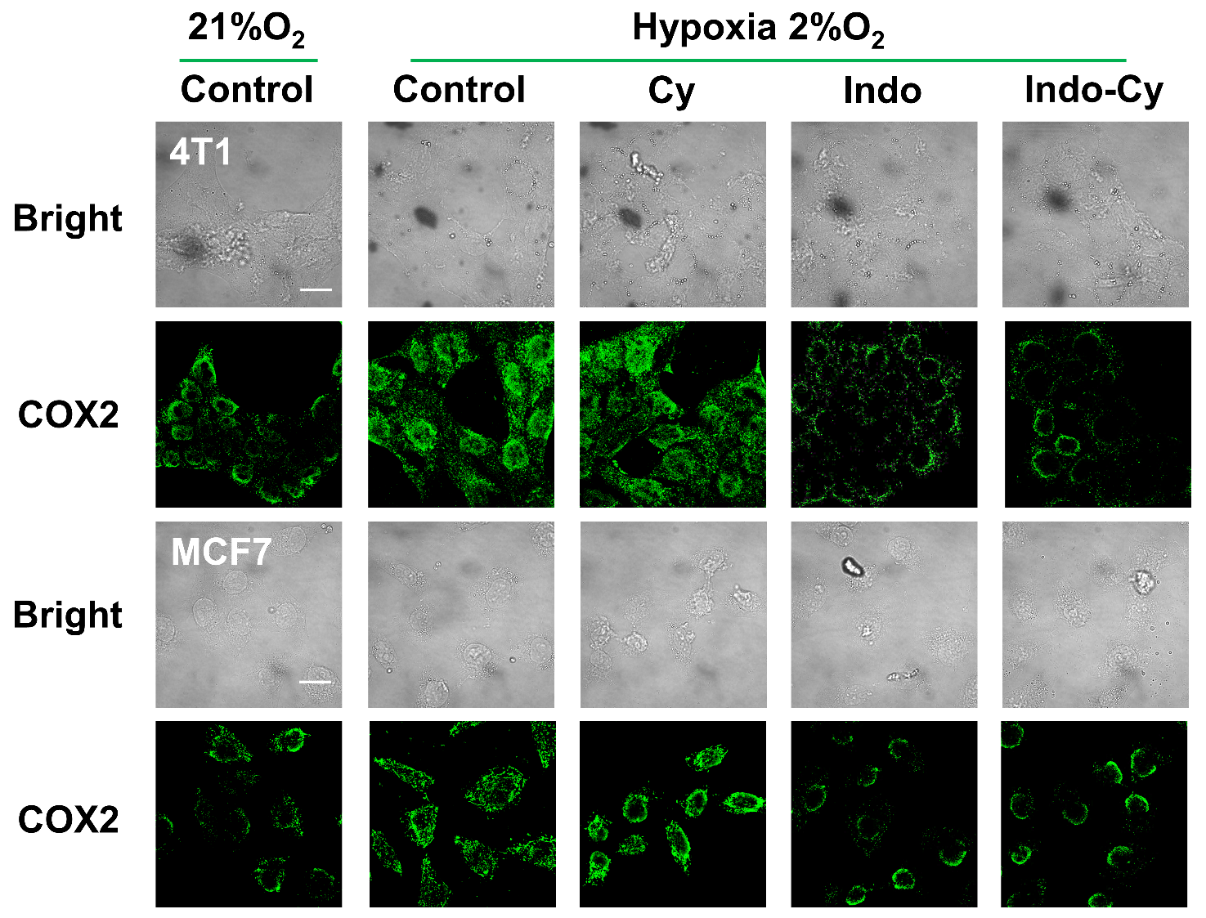


**Figure S16.** Immunofluorescent staining of COX-2 in 4T1 and MCF-7cells with different treatment. Scale bars: 20 µm.


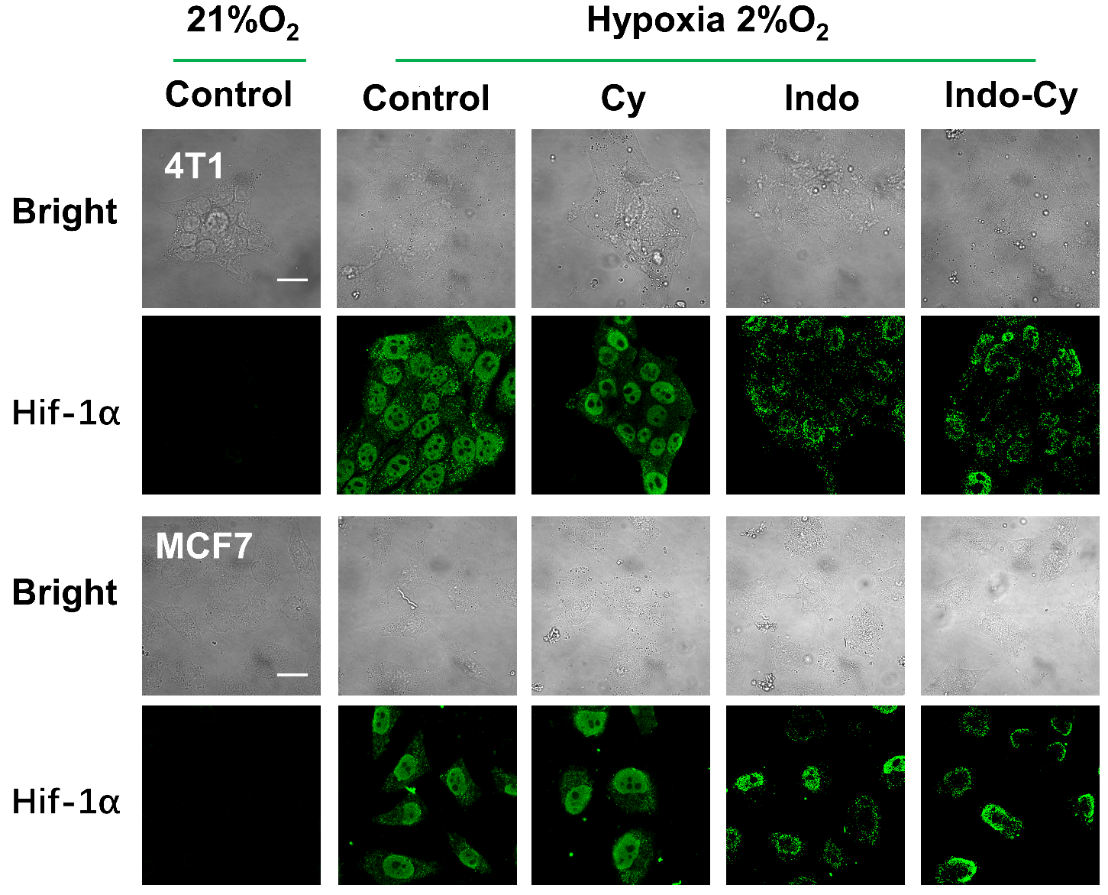


**Figure S17.** Immunofluorescent staining of HIF-1α in 4T1 and MCF-7cells with different treatment. Scale bars: 20 µm.


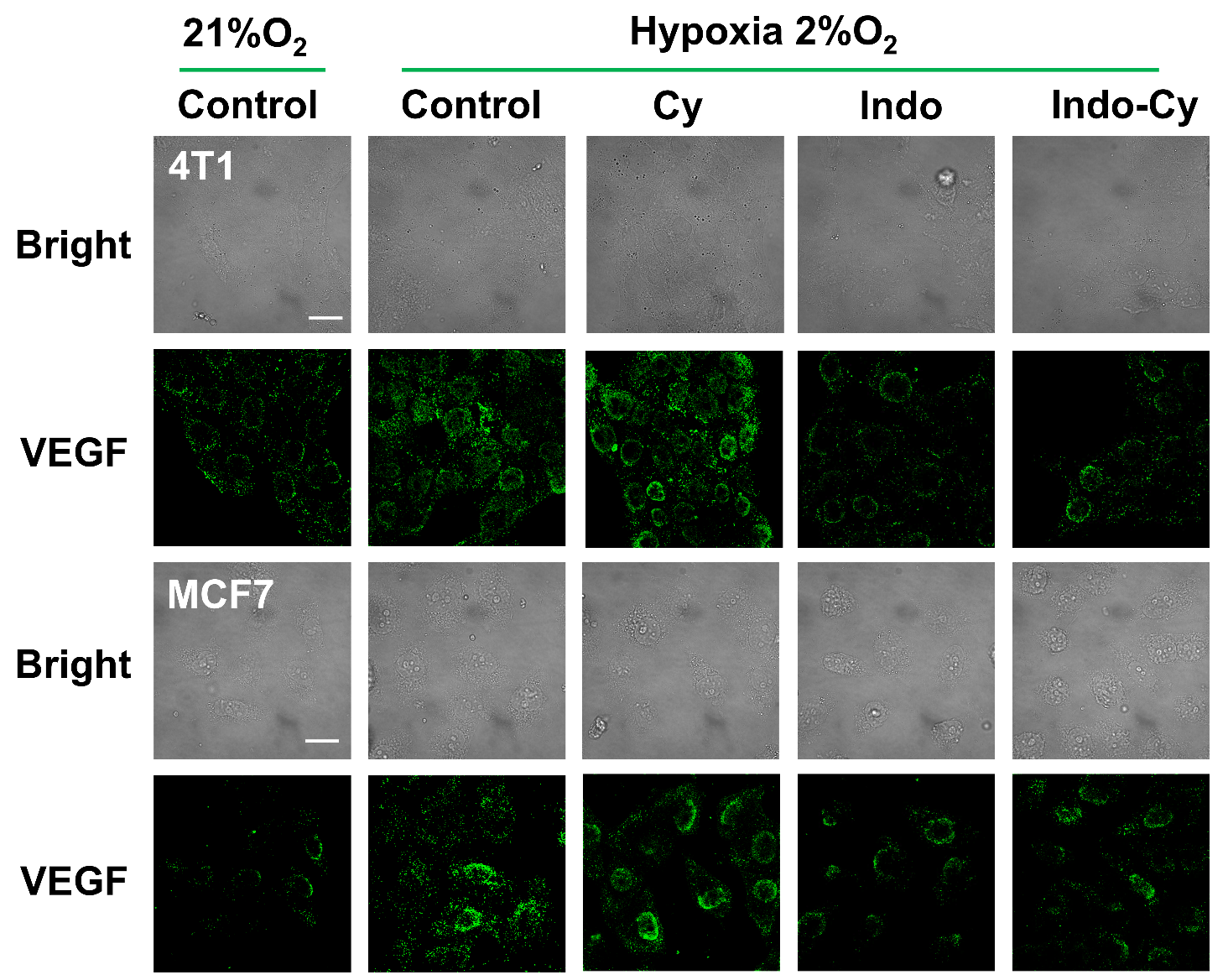


**Figure S18.** Immunofluorescent staining of VEGF in 4T1 and MCF-7cells with different treatment. Scale bars: 20 µm.


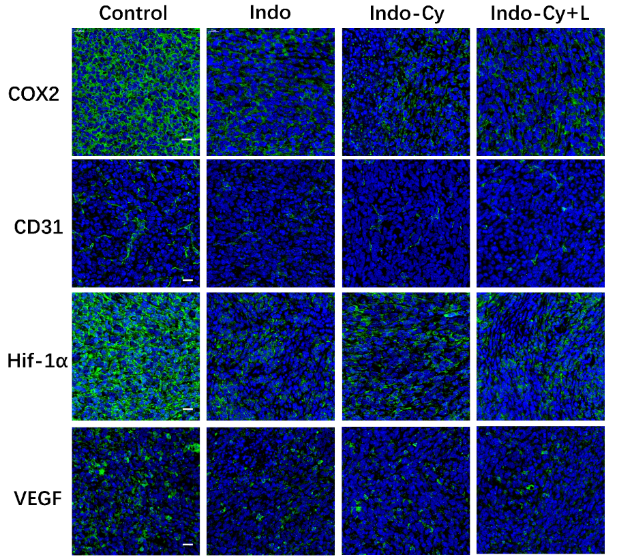


**Figure S19.** Immunofluorescent staining of COX-2, CD31, HIF-1α and VEGF in tumor tissues after treatment in different groups.


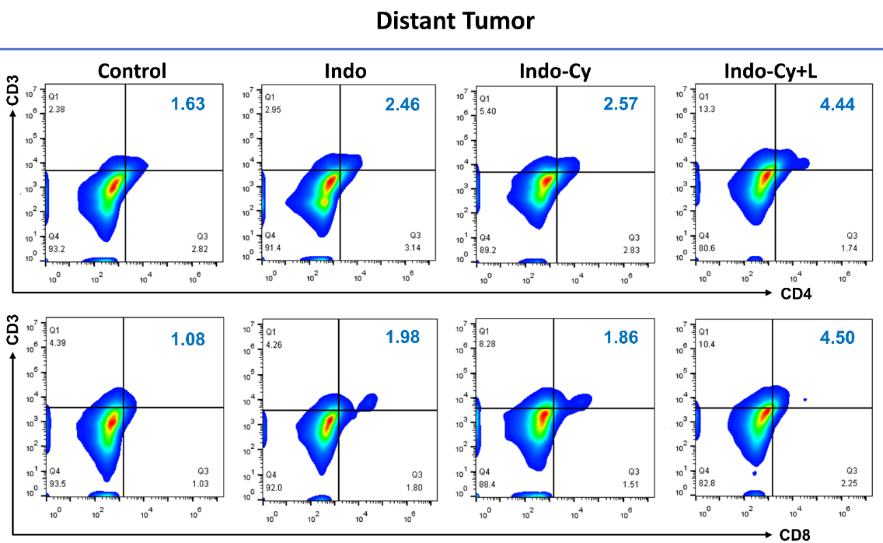


**Figure S20.** Representative flow cytometry images of CD4^+^ T cells and CD8^+^ T cells among CD3^+^ tumor-infiltrating leukocytes in the distant tumor tissues.


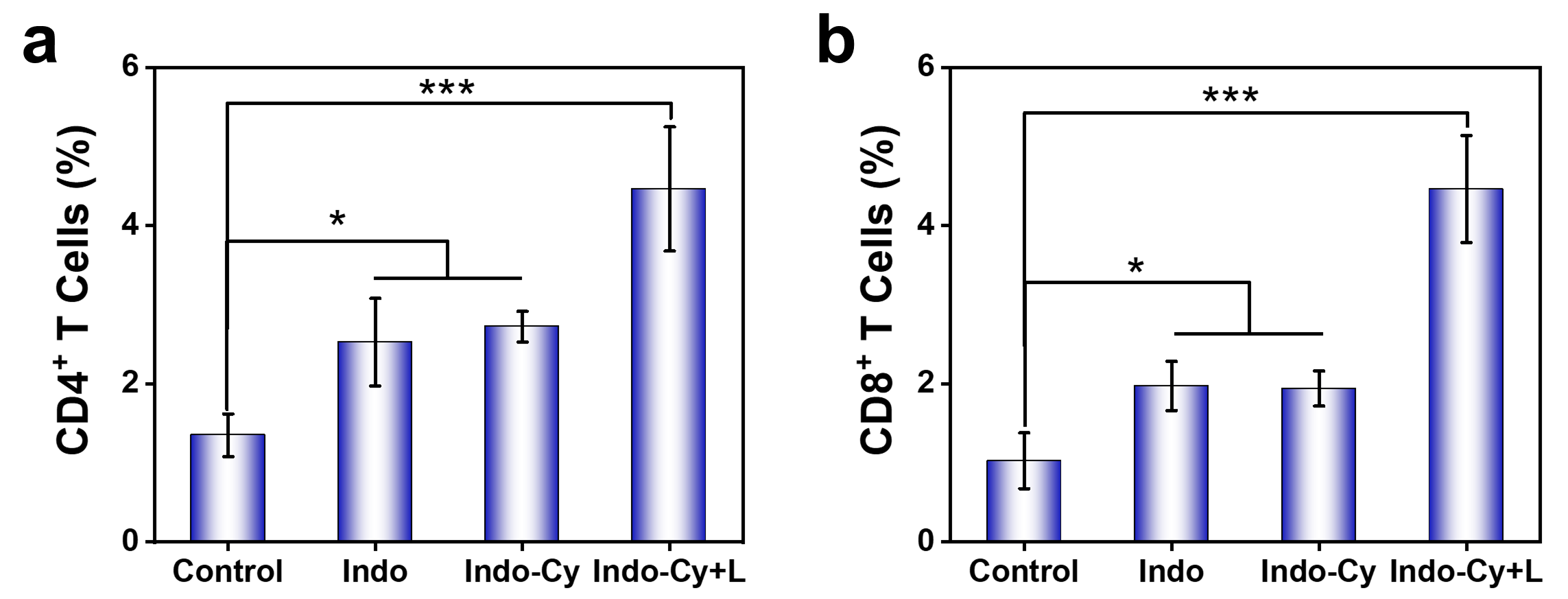


**Figure S21.** Quantification of the percentages of (a) CD4^+^ T cells and (b) CD8^+^ T cells in distant tumors based on flow cytometry.


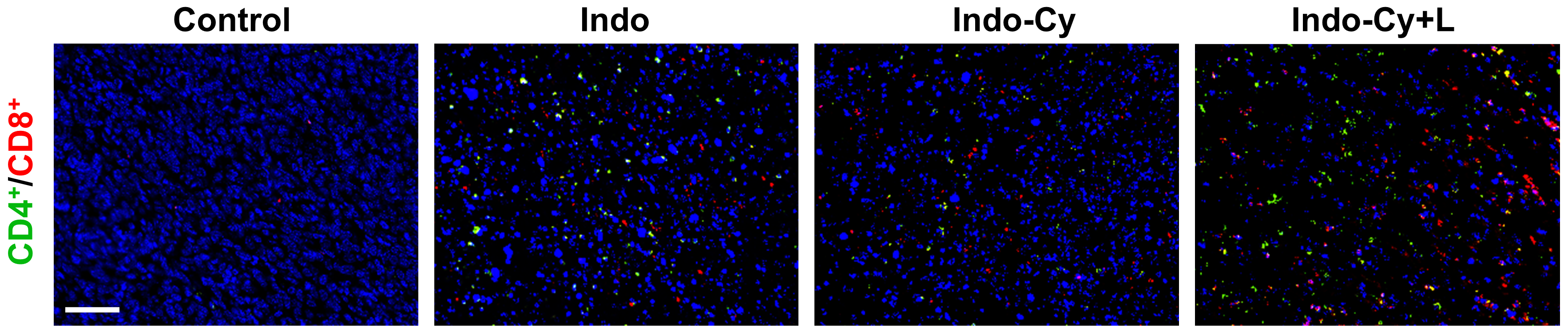


**Figure S22.** Immunofluorescence staining of CD4^+^ (green) and CD8^+^ (red) T lymphocytes in tumor tissues after different treatments. Scale bars are 40 μm.


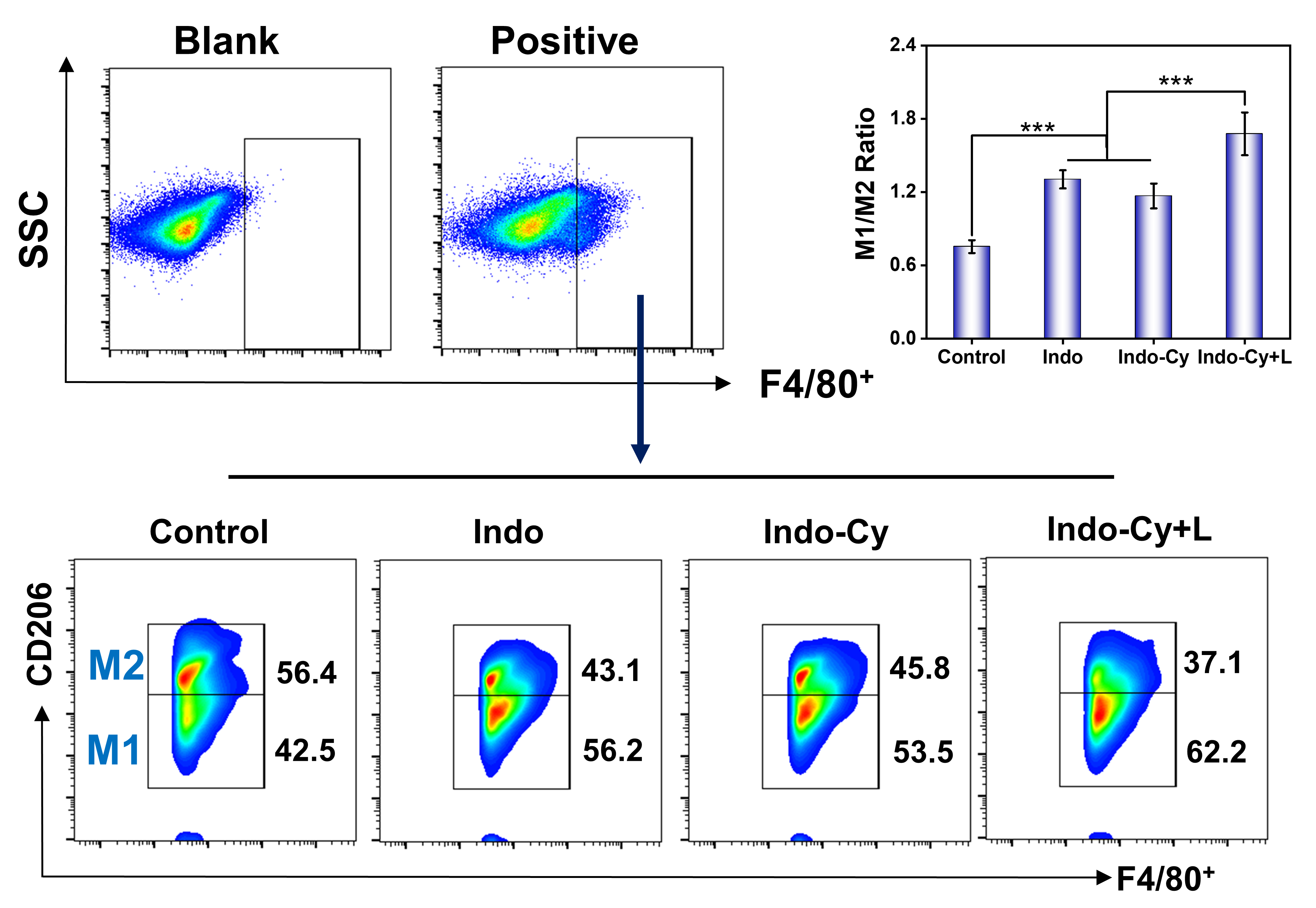


**Figure S23.** Representative flow cytometry images of M1 and M2 macrophages in primary tumor tissues.


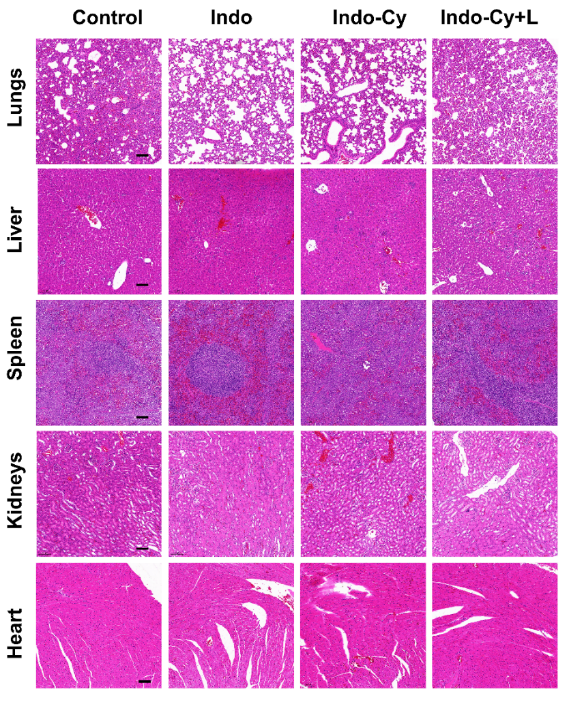


**Figure S24.** H&E staining of major organs of mice from different treatment groups after 14 d of treatment. Scale bar = 50 μm.


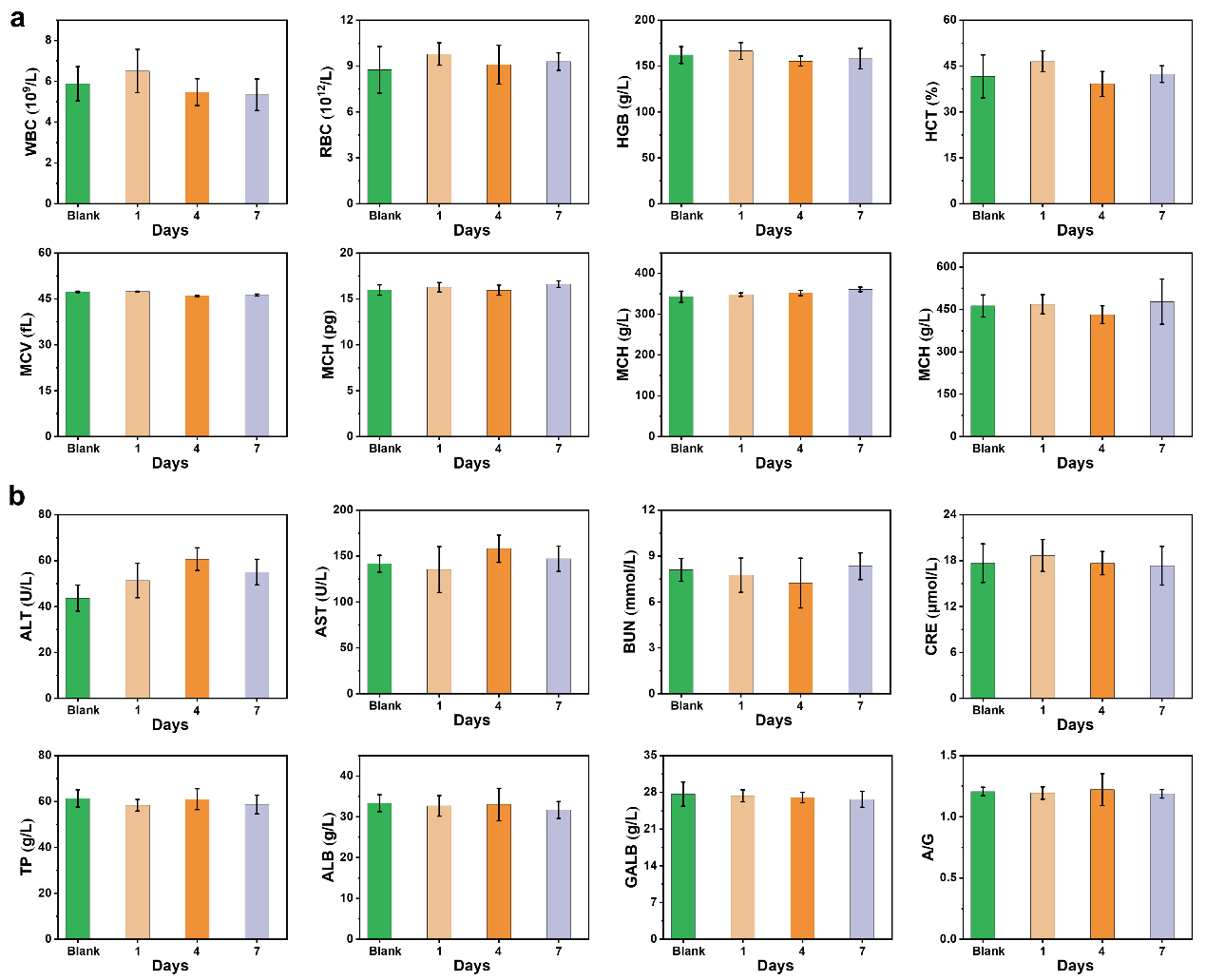


**Figure S25.** a) Hematological assessment conducted over a 7-day period post treatment, including WBC, RBC, HGB, HCT, MCV, MCH, MCHC, and PLT. b) Serum biochemical analysis for ALT, AST, BUN, CRE, TP, ALB, GLOB and A/G in 7 days. The error bars (n = 3) represent means ± SD.

References

1. Sharma, A., Lee, M.-G., Won, M., Koo, S., Arambula, J.F., Sessler, J.L., Chi, S.-G., and Kim, J.S. Targeting Heterogeneous Tumors Using a Multifunctional Molecular Prodrug. J. Am. Chem. Soc., 2019, *141*, 15611–15618.

2. Zeng, S., Chen, C., Zhang, L., Liu, X., Qian, M., Cui, H., Wang, J., Chen, Q., and Peng, X. Activation of pyroptosis by specific organelle-targeting photodynamic therapy to amplify immunogenic cell death for anti-tumor immunotherapy. Bioact. Mater., 2023, *25*, 580–593.

3. Zhang, Z., Xu, W., Kang, M., Wen, H., Guo, H., Zhang, P., Xi, L., Li, K., Wang, L., Wang, D., et al. An All‐Round Athlete on the Track of Phototheranostics: Subtly Regulating the Balance between Radiative and Nonradiative Decays for Multimodal Imaging‐Guided Synergistic Therapy. Adv. Mater., 2020, *32*, 2003210.

4. Becke, A.D. A new mixing of Hartree–Fock and local density-functional theories. The Journal of Chemical Physics, 1993, *98*, 1372–1377.

5. Andrae, D., Haubermann, U., Dolg, M., Stoll, H., and Preub, H. Energy-adjustedab initio pseudopotentials for the second and third row transition elements. Theoret. Chim. Acta 1990, *77*, 123–141.

6. Schäfer, A., Huber, C., and Ahlrichs, R. (1994). Fully optimized contracted Gaussian basis sets of triple zeta valence quality for atoms Li to Kr. The Journal of Chemical Physics *100*, 5829–5835. 10.1063/1.467146.

7. Tomasi, J., Mennucci, B., and Cammi, R. (2005). Quantum Mechanical Continuum Solvation Models. Chem. Rev. *105*, 2999–3094. 10.1021/cr9904009.

8. Lu, T., and Chen, F. (2012). Multiwfn: A multifunctional wavefunction analyzer. J. Comput. Chem. *33*, 580–592. 10.1002/jcc.22885.
